# Cross-Talk of Cation−π Interactions with Electrostatic and Aromatic Interactions: A Salt-Dependent Trade-off in Biomolecular Condensates

**DOI:** 10.1021/acs.jpclett.3c01642

**Published:** 2023-09-18

**Authors:** Milan Kumar Hazra, Yaakov Levy

**Affiliations:** Department of Chemical and Structural Biology, Weizmann Institute of Science, Rehovot 76100, Israel

## Abstract

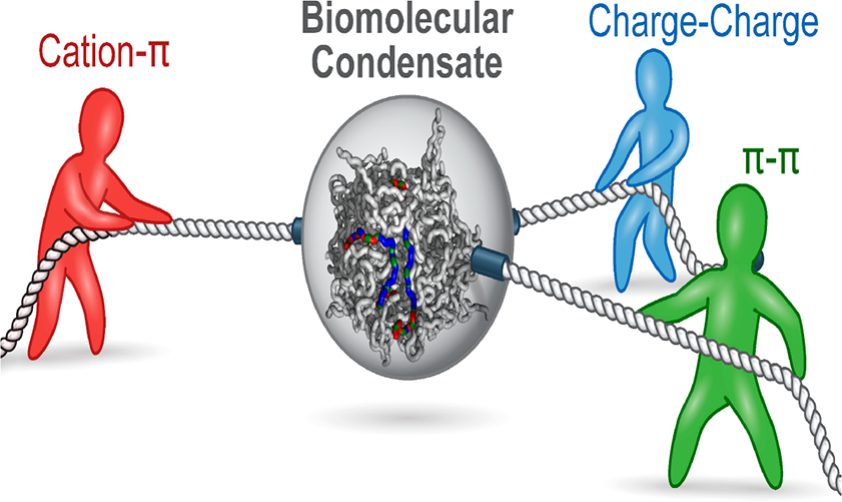

Biomolecular condensates are essential for cellular functionality, yet the complex interplay among the diverse molecular interactions that mediate their formation remains poorly understood. Here, using coarse-grained molecular dynamics simulations, we address the contribution of cation−π interactions to the stability of condensates formed via liquid–liquid phase separation. We found greater stabilization of up to 80% via cation−π interactions in condensates formed from peptides with higher aromatic residue content or less charge clustering. The contribution of cation−π interactions to droplet stability increases with increasing ionic strength, suggesting a trade-off between cation−π and electrostatic interactions. Cation−π interactions, therefore, can compensate for reduced electrostatic interactions, such as occurs at higher salt concentrations and in sequences with less charged residue content or clustering. Designing condensates with desired biophysical characteristics therefore requires quantification not only of the individual interactions but also cross-talks involving charge–charge, π–π, and cation−π interactions.

Biological mesoscale condensates have been found to regulate the functionality of some proteins and nucleic acids.^1−4^ Furthermore, anomalous protein condensate behavior has been shown to underlie several diseases and to arise from mutations affecting the specific amino acid sequences of the participating proteins and their interchain interactions.^5−9^ These findings have sparked interest in designing condensates to function as membraneless organelles to control cellular activities^10,11^ and to serve as therapeutic delivery agents^12^ and as biomaterials with tunable stability and dynamics.^10,12,13^ Liquid–liquid phase separation^14−18^ has emerged in the past decade as the primary mechanism by which biological systems attain such high-density membrane-less compartmentalization, producing condensates whose constituent proteins convert readily between their different conformations, engage in interactions through multivalent sticker motifs,^19,20^ and exhibit highly preserved mobility in liquid condensates.^17,21−23^

The genesis of biomolecular condensates, which are often formed by intrinsically disordered proteins (IDPs) that can be viewed as multivalent polymers,^24^ may be driven simply by long-range electrostatic interactions between charged residues in polyelectrolytic IDPs with high net charges (in the presence of oppositely charged polymers) or in polyampholytic IDPs.^25,26^ Typically, natural IDP sequences deviate from ideal polyelectrolyte or polyampholyte sequences, as they often comprise polar, aromatic, and hydrophobic residues. Indeed, not only charge–charge interactions,^25,27,28^ but also hydrogen bonding,^29^ π–π stacking,^30^ and cation−π^18,31,32^ interactions play crucial roles in the formation of biological condensates and in shaping their properties.

Several computational^33−37^ and experimental^38−43^ studies have endeavored to decipher the molecular grammar of the set of interactions that dominate condensate stability and dynamics. The interactions that drive the condensate phase may be classified as acting over short- or long-range intracellular distances. Earlier studies broadly defined a balance between the short- and long-range interactions that may stabilize the condensates, yet with diverging properties emerging depending on the sequence enrichment of these interactions.^44−46^ Although all nonelectrostatic interactions may be classified as short-range interactions as they act on a length scale of ∼1 nm, cation−π interactions^18,31^ are of special interest as they can be viewed as bridging between electrostatic and aromatic interactions.^47,48^ Recent experiments suggest the existence of multivalent cation−π interactions that play a dominant role in synaptophysin and synapsin coacervation^49^ and in Tau phase separation.^50,51^ Biomolecular condensates have been shown to retain their stability in extremely harsh conditions such as at very high salt concentrations.^52,53^ This observation indicates that nonionic interactions, namely, cation−π and π–π interactions, play a crucial role in these condensates, but their exact quantification is yet elusive. It has been postulated that IDP enrichment with Arg and Tyr often facilitates IDP recruitment in a preformed dense phase through cation−π interactions in addition to electrostatic interactions.^54,55^ FUS proteins undergo extensive phase separation due to the formation of cation−π interactions between the Arg and Tyr residues in their tails.^56^ We have shown that replacing charged residues with aromatic residues (and thereby replacing electrostatic interactions with π–π interactions while maintaining a net charge of zero) reduces condensate stability.^44^ In principle, mutating polyampholytes to aromatic residues may allow the formation of cation−π interactions in addition to π–π interactions. Although both cation−π and π–π interactions have a short-range nature, they may differ in their effect on condensate characteristics.

The current study aims to quantify the effects on condensate stability and dynamics that arise from cation−π interactions in the context of their trade-offs with other intracondensate interactions. Variation in the contribution and strength of the cation−π interactions may have different origins, including changes in the relative content of charged and aromatic residues, the organization of the charged residues, or the salt concentration of the solution. These changes in the IDP sequence or in the ionic strength of the solution may alter the content and strength of charged–charged and aromatic interactions, thus tuning potential cross-talks between them and affecting the formation of cation−π interactions (Figure 1A).

**Figure 1:**
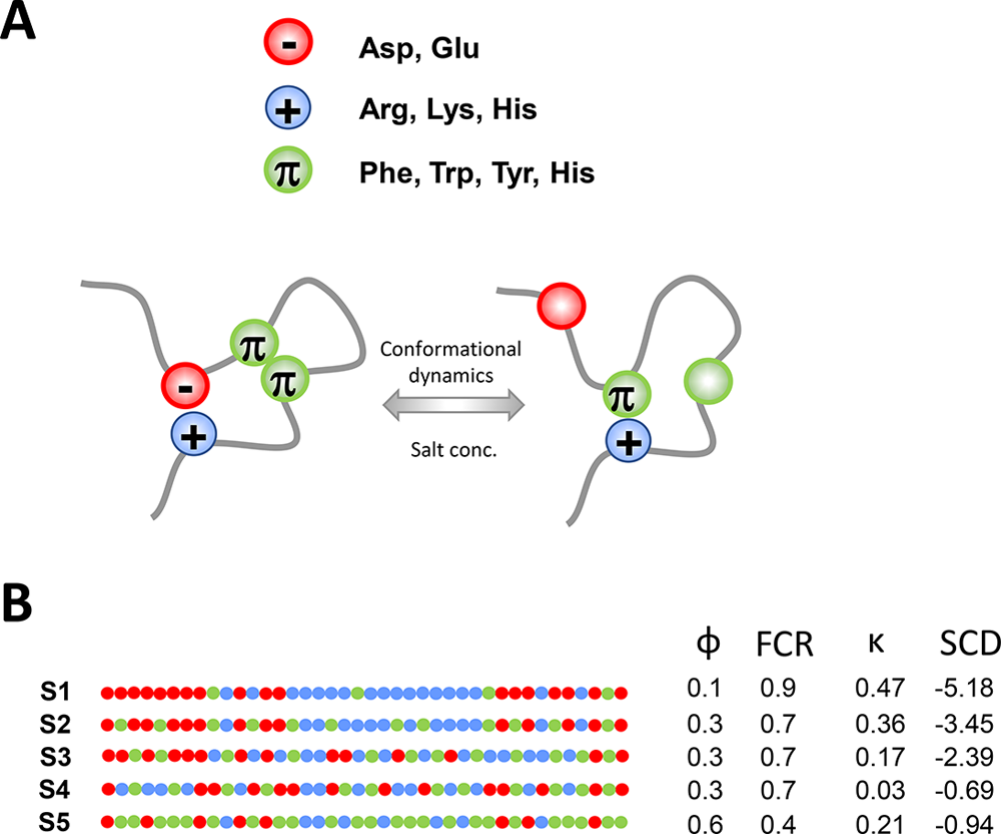
Interplay between charge–charge, π–π, and cation−π interactions within intrinsically disordered proteins that form biomolecular condensates. (A) A schematic illustration of frustration between purely electrostatic or aromatic interactions and cation−π interactions in an intrinsically disordered protein (IDP). The formation of a cation−π interaction may depend on breaking charge–charge and aromatic interactions, thereby making positively charged and aromatic residues available to interact with each other. This scenario may depend on the sequence characteristics and conformations of the IDPs, as well as on solvent salt concentrations, which modulate the overall strength of electrostatic interactions. Accordingly, cation−π, charge–charge, and π–π interactions in IDPs may not be additive, but rather exhibit compensatory behavior. (B) The five 40-amino acid sequences (designated S1–S5) designed for the current research to explore the interplay between cation−π, charge–charge, and π–π interactions. The peptides comprise three types of residues: positively charged (blue), negatively charged (red), and aromatic (green). Each sequence has a net charge of zero. The sequences differ in terms of their aromatic residue content (ϕ) and charged residue content (fraction of charged residues, FCR). Some of the sequences also have different charged residue clustering coefficients, κ, which was achieved by shuffling the residues within the sequence. Sequences S2–S4 have identical numbers of charged and aromatic residues but differ with respect to their organization, as reflected by their different κ values. Sequences S2 and S5 were obtained from the S1 sequence by gradually replacing its charged residues with aromatic residues (which reduces FCR and, consequently, the value of κ).

The formation of cation−π interactions can be indirectly regulated by changing solvent salt concentration, which tunes the prevalence of electrostatic interactions in the sequences. For a more direct approach, it is known that manipulating the content and organization of charged residues can influence the balance between their short- and long-range interactions as well as their stability,^44^ which consequently may directly affect their propensity to form cation−π interactions. Therefore, to gain understanding into the complex blending of long-range and short-range interactions between IDPs in biomolecular condensates, we designed five IDP sequences (designated S1–S5; Figure 1B) that differed with respect to their aromatic residue content (ϕ), their charged residue content (as indicated by the fraction of charged residues, FCR), and/or their charged residue clustering coefficients (κ)^57^ and/or sequence charge decoration in the sequences (SCD).^58^ IDPs S1–S5 comprise 40 residues of which 4 (S1) to 24 (S5) residues are aromatic, such that aromatic residue content ranges from 0.1 (S1) to 0.6 (S5) and FCR is correspondingly 0.9 (S1) to 0.4 (S5). All five designed sequences have a net charge of zero and are composed of only three types of residues: positively charged, negatively charged, and aromatic residues. The number of positively and negatively charged residues in the sequences ranges from 18 (S1) to eight (S5), with S2 and S4 all having 14 positively and negatively charged residues but differing in terms of how their charged residues are organized along the sequence. Differences in charged residue organization manifest in different charged residue clustering coefficients, κ, which have an overall range of κ = 0.47–0.21 for S1–S5 and of κ = 0.36–0.03 for S2–S4 (see Figure 1B).

A coarse-grained molecular dynamics model was employed to simulate multiple copies of each of the designed sequences to study their self-condensation in an aqueous solution. Each residue in each of the five 40-residue sequences was modeled as a bead that could be positively charged, negatively charged, or have aromatic characteristics (see Figure 1).

The potential energy function consists of several terms: bonded and angular terms, long-range electrostatic interactions among all charged beads, both intra- and intermolecularly, and short-range dispersion interactions. Short-range dispersion interactions are applied to model interactions between the aromatic residues (*i.e*., π–π interactions) and between aromatic beads and positively charged beads (*i.e*., cation−π interactions). In addition, an excluded volume potential is applied to all bead pairs. The bonded and angular interactions are modeled with a harmonic potential. As IDPs have high conformational flexibility, which is important in the formation of biological network-based condensates, the studied sequences are modeled as completely flexible without any dihedral angle constraint.

The electrostatic interactions between the charged beads are defined using the Debye–Hückel potential^44,59,60^*E*_electrostatic_ =  where *q*_*i*_ and *q*_*j*_ denote the charge of the *i*^th^ and j^th^ bead, *r*_*ij*_ denotes the interbead distance, ε is the solvent dielectric constant, and *K*_Coulomb_ = 4*πε*_0_ = 332 kcal/mol. *B*(κ_D_) is a function of solvent salt concentration and the radius (*a*) of ions produced by the dissociation of the salt, and if can be expressed as . The Debye–Hückel electrostatic interactions of an ion pair act over a length scale of the order of , which is called the Debye screening length. The Poisson–Boltzmann equation leads to the following relation of κ_D_ to ionic strength:  where *N*_A_ is the Avogadro number, *e* is the charge of an electron, *ρ*_A_ is the solvent density, *I* denotes solvent ionic strength, *k*_B_ is the Boltzmann constant, and *T* is the temperature. The short-range dispersion interactions between a pair of aromatic beads (*i.e*., π–π) or between an aromatic bead and a positively charged bead (*i.e*., cation−π) are modeled using the Lennard-Jones potential: *E*_π–π/cation−π_ =  where *σ*_*ij*_ denotes the optimal distance between beads *i* and *j* that are in contact with each other and *σ*_*ij*_ = 7 Å. The parameter ε denotes the strength of the short-range dispersion interaction. We used ε = 0.2 kcal/mol, as this value was shown to reproduce the experimentally determined radius of gyration (Rg) of several IDPs as estimated from their simulations using a coarse-grained model that included only electrostatic and hydrophobic interactions.^44^ Given that cation−π interactions in proteins are of similar strength to π–π interactions,^61,62^ we modeled both types of interactions at the same epsilon value (*i.e*., ε_π–π_ = ε_cation−π_ = 0.2 kcal/mol). Our model assumes that the cation−π interaction strength is independent of the salt concentration. We have observed that the same cation−π interaction strength reproduces their experimentally determined Rg values for two different salt concentrations (i.e., 20 and 40 mM). In addition, we heuristically calibrated the cation−π interaction strength to reproduce experimental Rg of these IDPs. We observed that cation−π interaction strength of 0.2 kcal/mol along with aromatic strength of 0.2 kcal/mol serves best to reproduce highest correlation between experimental and simulated Rg’s (Figures S1–S4). We note that while ions may play an important role in determining the thermodynamic stability of the condensate phase,^26,63^ our model assumes that once the condensates are formed nearly all counterions are released to the bulk. Accordingly, the probability of competition between the residue–residue cation−π and ion–residue cation−π within a condensate phase seems to be low unless there is a significant trapping of ions in condensates.

To examine cross-talks between cation−π aromatic stacking interactions, we also simulated a selected subset of systems at a higher cation−π strength (ε_cation−π_ = 0.3 kcal/mol while ε_π–π_ = 0.2 kcal/mol). In order to avoid overlap between beads, each bead interacts with all other beads through a steep repulsion interaction defined by *E*_repulsion_ = with *σ*_*ij*_ = 4 Å. The initial configuration of the studied IDPs was designed by randomly placing 100 copies of each studied ID in a box with dimensions of 300 × 300 × 300 Å. Multiple copies for each of the simulations were performed by solving the Langevin equation to achieve proper averaging at several temperatures and salt concentrations. A clustering algorithm was used to distinguish the individual condensates and their component polymers at each time step.

To study the interplay among cation−π, charge–charge, and aromatic π–π interactions between IDPs in biomolecular condensates, we independently studied 100 copies of each of the five sequences S1–S5. Each system was studied by using two related coarse-grained molecular dynamics models. Both models allowed the peptides to take part in electrostatic (charge–charge) and aromatic (π–π) interactions, but they differed regarding whether they included or excluded the formation of cation−π interactions. Each system was studied at different solution salt concentrations to manipulate the strength of the electrostatics interactions and thereby allow for examination of their cross-talks between all three types of interactions potentially occurring within the condensate. At each salt concentration, each of these systems was simulated over a large temperature range that covers its transition from weakly interacting free chains to a condensed assembly of highly interacting chains. These simulations were used to construct phase diagrams for liquid–liquid phase separation in each system, with the concentration of the dilute and dense phases plotted for different temperatures.

In Figure 2, the left and middle panels show phase diagrams (temperature *T* versus solvent density ρ) for selected peptide systems, highlighting the critical temperature, *T*_C_, beyond which separation does not occur and therefore refers to the stability of the condensate. Each system was studied under both the cation−π-inclusive (filled symbols) and cation−π-exclusive models. The arrows in the phase diagrams indicate *T*_C_ and are positioned to show the difference in *T*_C_ (Δ*T*_C_) between these two models. Figure 2 (right) summarizes the dependence of Δ*T*_C_ on some key molecular features of the systems, as discussed below.

**Figure 2:**
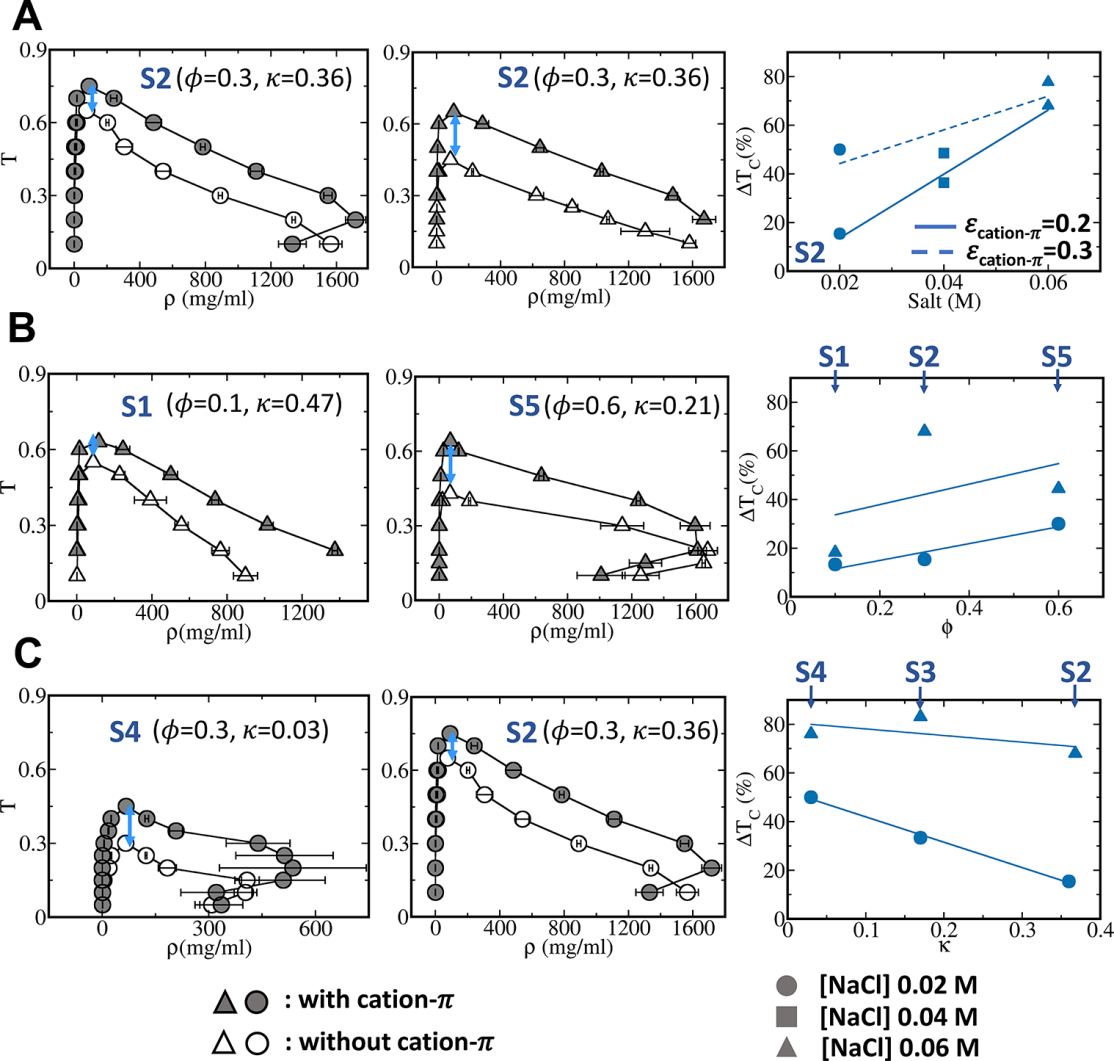
Effects of cation−π interactions on the stability of peptide condensates having different properties. The effect of cation−π interactions is explored as a function of salt concentration of the solution (A); the aromatic residue content, ϕ, of the sequence (B); and the charge clustering coefficient, κ, of the sequence (C). Representative phase diagrams for the liquid–liquid phase separation of selected peptides are plotted (left and middle panels). Each peptide was simulated using coarse-grained models that include (filled symbols) and exclude (empty symbols) cation−π interactions at NaCl concentrations of 0.02 M (circles) and 0.06 M (triangles). For each temperature in a phase diagram, an error bar has been shown as the standard deviation obtained from three independent simulation trajectories. Critical temperature has been obtained by fitting density–temperature data with the ρ_dense phase_ – ρ_dilute phase_ = , where *ρ*_dense phase_ and *ρ*_dilute phase_ denote the polymer bead density in the dense phase and in the dilute phase, respectively, and β is a critical exponent that, following other studies, was set to 0.325. The estimation of critical temperature has also been validated by a change in slope of condensate phase density along temperature. The effect of cation−π interactions on the stability of each peptide condensate is indicated by the change in the critical temperatures (*T*_C_) obtained from simulations performed using the cation−π-inclusive and cation−π-exclusive models (*i.e*., Δ*T*_C_ = *T*_C(incl_cation−π)_ – *T*_C(excl_cation−π)_). Δ*T*_C_ is schematically illustrated in each phase diagram by the blue arrows. The right panels summarize the relationship between normalized Δ*T*_C_ (in %) and salt concentration, κ, and ϕ. The dependence of Δ*T*_C_ on salt concentration is examined for two cation−π interaction strengths ((ε_cation−π_ = 0.2 or 0.3 kcal/mol). The linear fit is shown for each of these correlations (dashed or solid lines). The peptides discussed in each panel are indicated on it. At moderate and high aromatic content, we have observed a re-entrant phase behavior with condensate phase density being lowered as temperature is decreased. This observation stems from the fact that short-range interactions are dominant in this regime, and as temperature is lowered, kinetic arresting may play an important role once electrostatic interactions are diminished in conjunction with lowering temperature.

To study the interplay between cation−π interactions and salt concentration, we studied the S2 peptide at three salt concentrations (0.02 0.04, and 0.06 M). We selected the S2 peptide for this purpose as it has a medium aromatic residue content (ϕ = 0.3; *i.e*., 30% of its residues are aromatic), which indicates that positively charged and aromatic residues are present in almost equal proportions and so maximizes the potential for cation−π crosstalk, and a medium charge clustering coefficient (κ = 0.36) (see Figure 1A). Figure 2A shows that as the salt concentration increases from the left to middle panel, condensate stability, as indicated by the value of *T*_C_, decreases when using the cation−π-exclusive model (empty symbols), as expected from salt-related weakening of electrostatic interactions. The stability of the condensates simulated using the cation−π-inclusive model (filled symbols) also decreases as salt concentration increases, but only slightly. The finding that the stability of the condensates is much less sensitive to salt concentration under the cation−π-inclusive model indicates that the formation of cation−π interactions compensates for the loss of stability due to loss of electrostatic interactions. A 3-fold increase in salt concentration enhances condensate *T*_C_ by 15–60% (Figure 3A, right panel) because of the formation of cation−π interactions. The increase in Δ*T*_C_ at higher salt concentrations reflects a trade-off between electrostatic and cation−π interactions.

**Figure 3:**
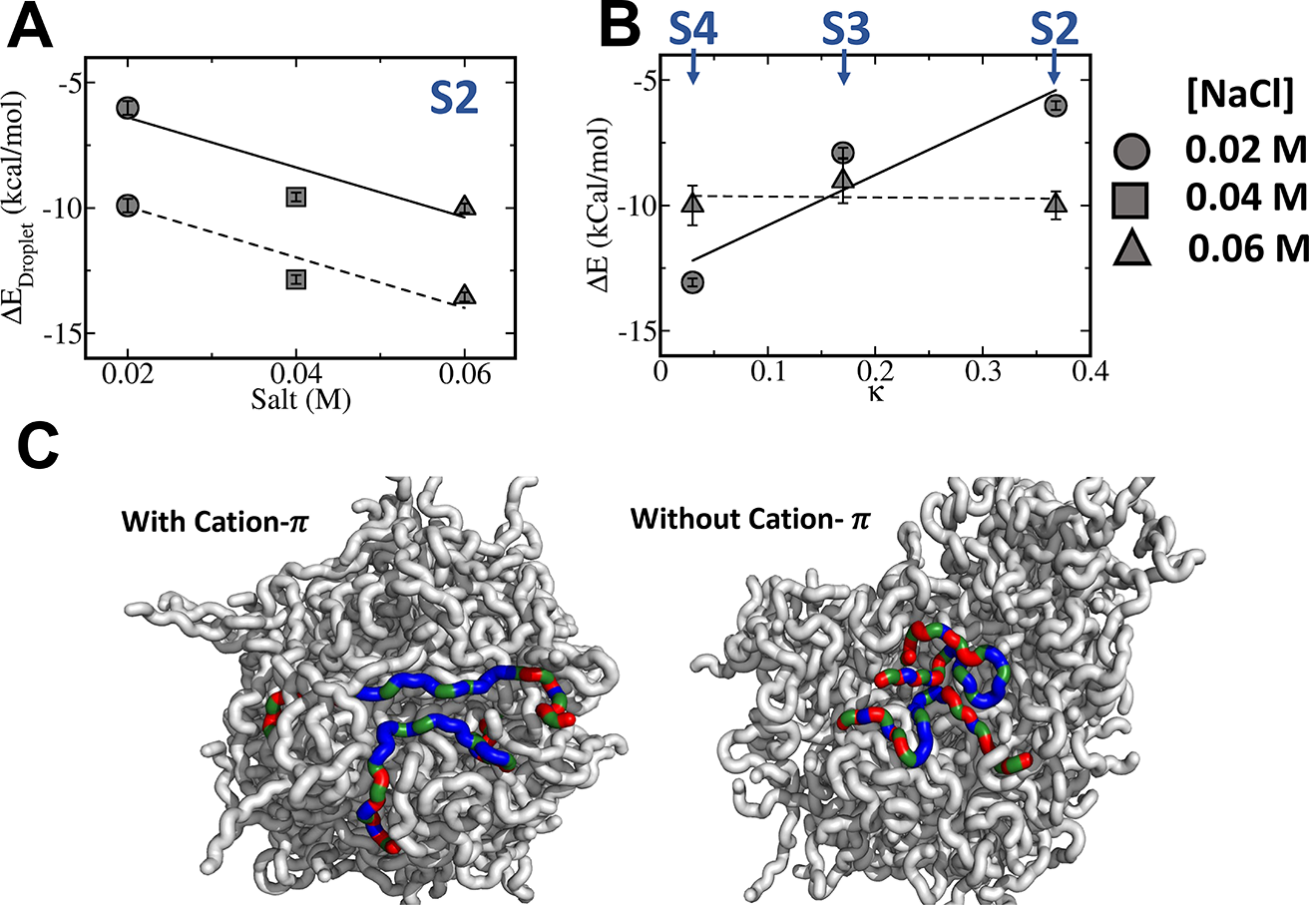
The energetic gain due to cation−π formation in biomolecular condensates. The energetic gain, Δ*E*_Droplet_, is estimated as the energy gained by each polymer within the condensate using the cation−π-inclusive model. (A) The energetic gain due to cation−π interactions is shown for peptide S2 simulated at different salt concentrations and for two cation−π interaction strengths (ε_cation−π_ = 0.2 or 0.3 kcal/mol, fitted to solid and dashed lines, respectively). (B) The energetic gain due to cation−π interactions for peptides S2–S4, which differ with respect to charge clustering (κ). Changing κ can reduce cation−π stabilization; however, this effect strongly depends on salt concentration. At a salt concentration of 0.02 M, the energetic gain arising from the formation of cation−π interactions varies by 6–13 kcal/mol depending on the κ value. At a higher salt concentration of 0.06 M, characterized by weak electrostatic interactions, the energetic gain from cation−π interactions is independent of variation in κ and each polymer gains nearly 10 kcal/mol energetic stabilization in the condensate phase under the cation−π-inclusive model compared with the cation−π-exclusive model. (C) Illustrative snapshots of two S2 polymers interacting within a condensate droplet formed at a salt concentration of 0.06 M and *T*/*T*_c_ = 0.4 and modeled using the cation−π-inclusive (left) and cation−π-exclusive models. In these snapshots, positively and negatively charged residues are colored blue and red, respectively, and the aromatic residues are shown in green.

The trade-off is illustrated by the droplet gaining energy at higher salt concentrations despite the breakage of electrostatic interactions. The formation of cation−π interactions in the dense phase results in an energetic gain of ∼6–10 kcal/mol per peptide at salt concentrations of 0.02–0.06 M (Figure 3A). This increase in stability arising from the formation of cation−π interactions was obtained using a model that assumes identical strengths for cation−π and π–π interactions (*i.e*., ε_π–π_ = ε_cation−π_ = 0.2). To examine the effect of even stronger cation−π interactions on Δ*T*_C_, we simulated the S2 peptide for ε_cation−π_ = 0.3 (Figure 2A, upper values, dashed line), which enhanced the stability of its condensate to ∼50% and ∼80% at low and high salt concentrations, respectively (Figure 2A). For the stronger cation−π interactions, the energetic stabilization per polymer in the condensate increases to be between 10 and 14 kcal/mol depending on the salt concentration (Figure 3A, dashed line).

Next, we examined the effect of aromatic residue content, ϕ, on the formation of cation−π interactions in the condensate and thus on stability, *T*_c_. We note that a change in ϕ is naturally coupled with a change in the charged residue content and therefore also changes the κ value. The interplay between ϕ and *T*_c_ occurring during the formation of cation−π interactions is examined by studying the phase separation of sequences S1, S2, and S5, which have ϕ values of 0.1–0.6 (i.e., FCR values of 0.9–0.4; see Figure 1). Figure 2B (right panel) shows that, using the cation−π inclusive model and at salt concentration of 0.02 M (filled round symbols), the gain in stability of the condensate phase is higher for ϕ = 0.6 (sequence S5; Δ*T*_C_ = 30%) than for ϕ = 0.1 (sequence S1; Δ*T*_C_ = 10%). The value of Δ*T*_C_ for condensates of these sequences including and excluding cation−π interactions is 20–45%, respectively, when simulated at a higher salt concentration of 0.06 M. The condensate phase is significantly less dense when cation−π interactions are excluded rather than included, and the difference in critical temperature between systems simulated with and without cation−π interactions is enhanced for higher aromaticity content. The enhancement of the stability of the droplet phase that is contributed by cation−π interactions for higher ϕ values is greater at higher salt concentrations than at lower salt concentrations (Figure 2B). When aromatic content is lower (ϕ = 0.1), electrostatics play the dominant role, whereas when aromatic content is higher (ϕ = 0.6) the aromatic interactions dominate. However, at an intermediate aromatic content (ϕ = 0.3) we observe maximal energetic stabilization (Δ*T*_C_ ≈ 70% compared with Δ*T*_C_ values of 20% and 45% for ϕ = 0.1 or 0.6, respectively), which is achieved via cation−π crosstalk with the charge–charge and π–π interactions (Figure 2B and Figure S7).

The observed interplay between salt concentration and ϕ concerning the extent of formation of cation−π interactions (with consequent effects on Δ*T*_C_), suggests that variations in the charged residue clustering patterns (represented by κ) may also modulate cation−π interactions, as κ affects electrostatic interactions. To estimate the existence of trade-offs between cation−π and κ, we examined the properties of the condensates formed by sequences S2 and S4, which have identical charged and aromatic residue contents (ϕ = 0.3, which also ensures a potentially high number of cation−π interactions) and with κ of 0.03–0.36 (see Figure 1B). Figure 2C (right panel) shows the correlation between Δ*T*_C_ and κ for two different salt concentrations of 0.02 (circles) and 0.06 M (triangles). As κ increases, the cation−π interactions have a reduced effect on the stability of the condensate, as indicated by the strong negative correlation between Δ*T*_C_ and κ at a salt concentration of 0.02 M. Figure 2C shows a Δ*T*_C_ of ∼50% for the S4 peptide with κ = 0.03 compared with Δ*T*_C_ of ∼15% for the S2 peptide with κ = 0.36. As charge clustering in the sequences decreases (*i.e*., at lower κ values), more electrostatic interactions are disrupted, which enables more positively charged residues to engage in cation−π interactions. Consequently, cation−π interactions have less of an effect for sequences with higher κ values. This trend depends on salt concentration. As electrostatic interactions weaken as salt concentration increases, the effect of charge clustering (*i.e*., of the κ value) on the electrostatic energy also reduces. Consequently, at a salt concentration of 0.06 M, Δ*T*_C_ exhibits weaker dependence on κ than that observed at a salt concentration of 0.02 M. At salt concentrations of both 0.02 and 0.06 M (Figure 2B; right panel) Δ*T*_C_ is positive, yet Δ*T*_C_ is greater at the higher salt concentration, consistent with Figure 2A (right panel).

The relation between salt concentration, κ value, and the ability to form cation−π interactions is supported by the energetic analysis (Figure 3B). It is evident that, at a low salt concentration of 0.02 M (Figure 3B, circles), the energy of each polymer decreases by ∼13 kcal/mol as a result of cation−π interactions with neighboring sequences in the droplet for sequences with low κ values (*i.e*., S4) compared with a condensate having no cation−π interactions, whereas the additional stabilization per polymer is only ∼6 kcal/mol for sequences with higher κ values (*i.e*., S2) (Figure 3B). At a higher salt concentration of 0.06 M, the energetic gain per polymer in the droplet phase is much less sensitive to κ and remains nearly uniform at ∼10 kcal/mol. Figure 3C shows illustrative snapshots of S2 droplets formed with and without cation−π interactions, respectively, as indicated by interactions between blue and green residues, which occur only in one case.

The incorporation of cation−π interactions in the condensate, which in all the cases studied here increased condensate stability, is expected to reduce internal diffusion by individual polymers within the droplet. However, given the sensitivity of the energetic contribution of the cation−π interactions to the molecular properties of the constituent peptides, the effect of cation−π interactions on the liquidlike nature of the droplet may also depend on these molecular properties. To quantify the effect of cation−π interactions on peptide diffusivity in the condensate phase, we calculated the difference between the extent of internal diffusion under the cation−π-inclusive and cation−π-exclusive models. In order to focus on the effect of cation−π interactions on translational diffusion in the dense phase, we normalized the translational diffusion coefficient of each peptide in the droplet by its diffusion coefficient in the bulk (*D*_droplet_/*D*_bulk_). Accordingly, the effect of cation−π interactions on translational diffusion is probed by the difference between the two *D*_droplet_/*D*_bulk_ ratios for systems in which cation−π interactions are included or excluded (Figure 4). The diffusion constants were obtained from the slope of the linear fit to the mean squared displacement (MSD) of the polymer in the dense phase or in the bulk. MSD in three dimensions is related to the diffusion constant as follows, *D* = . When the diffusion coefficient in the dense phase was calculated, we ensured that the peptides were part of the condensate for the entire time window of interest.

**Figure 4:**
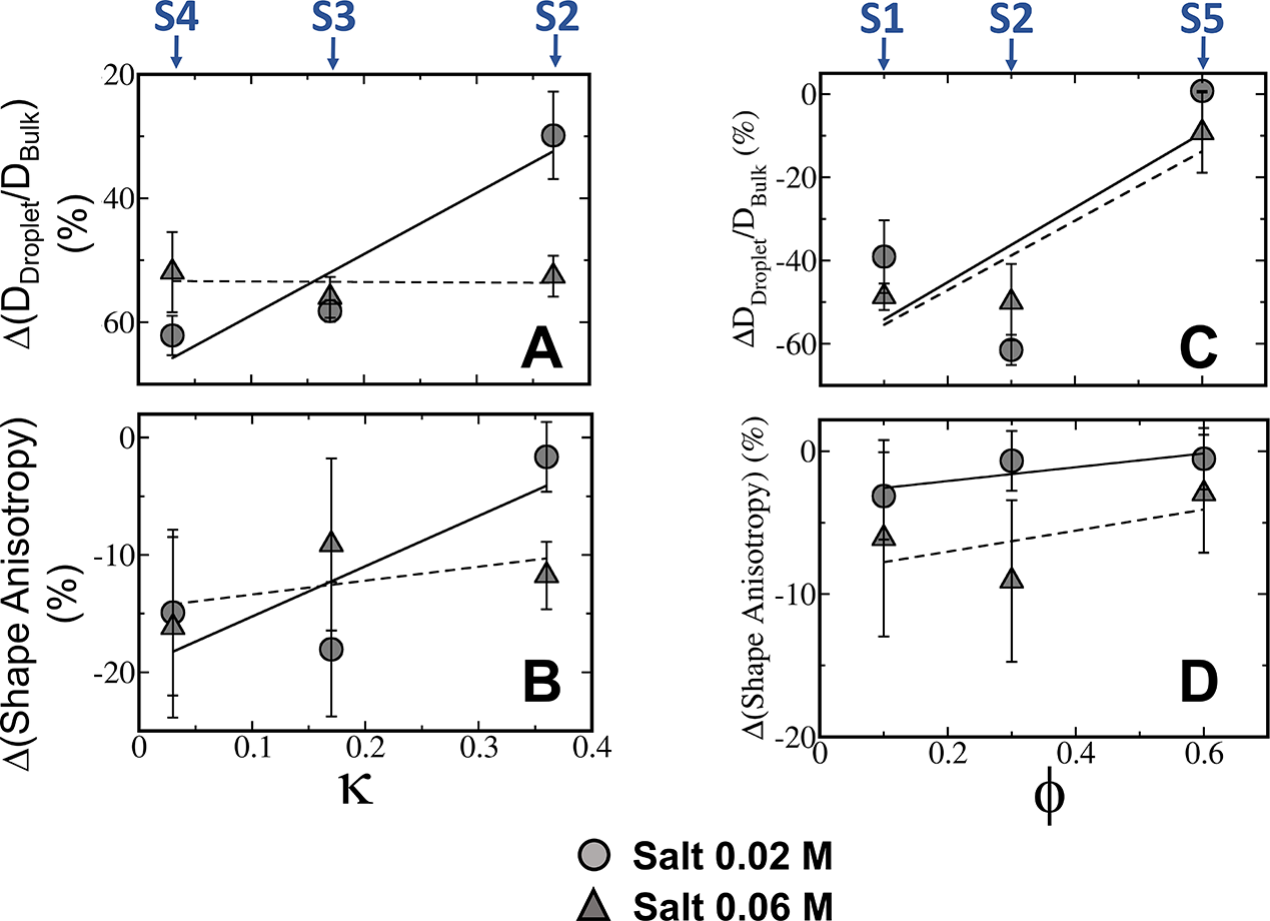
Cation−π interactions influence the shape and liquid-like nature of peptide condensates. The change in diffusivity (A and C) and shape anisotropy (B and D) for condensates simulated using the cation−π-inclusive and cation−π-exclusive models as a function of charge clustering (κ) (for peptide sequences S2–S4; panels A and B) and aromatic residue content (ϕ) (for peptide sequences S1, S2, and S5; panels C and D) at salt concentrations of 0.02 M (circles, fitted with solid line) and 0.06 M (triangles, fitted with dashed line). The change in diffusivity is calculated as the difference of the ratio *D*_Droplet_/*D*_Bulk_ obtained using the cation−π-inclusive and cation−π-exclusive models.

For peptides S2 and S4 that have identical aromatic residue contents of ϕ = 0.3, the diffusivity of the peptides in the condensate phase is observed to reduce when cation−π interactions are included as κ reduces from 0.36 to 0.03 at a low salt concentration of 0.02 M (Figure 4A; circles). For example, a ∼60% lower normalized diffusion coefficient is measured in condensates with κ = 0.03, compared with a reduction of ∼30% for peptides with κ = 0.36. This reduced diffusivity due to cation−π interactions in condensates formed by peptides of low κ is not observed at the higher salt concentration of 0.06 M (Figure 4A; triangles).

The different manifestation of the cation−π interactions on the liquid nature of peptides S2 and S4 at low and high salt concentrations is consistent with the difference in energetic gain of these systems at these salt concentrations (Figure 3B). The electrostatic interactions are weaker under conditions of higher compared with lower salt concentration, which makes sufficient positively charged residues available to participate in cation−π interactions. The participation of more positively charged residues in cation−π interactions at the high salt concentration of 0.06 M enhances condensate stability through an energetic gain of approximately −10 kcal/mol per monomer (Figure 3B) and reduces peptide diffusivity by ∼50% (Figure 4A, left panel), for various κ values.

The gradual replacement of electrostatic residues by aromatic residues, leading to increased aromatic residue content ϕ (as observed in the series of peptides S1, S2, and S5) increases the diffusivity of the corresponding condensate phase of these peptides when cation−π interactions are included. The maximal loss of diffusivity of the condensate phase upon inclusion of cation−π interactions (compared with their exclusion) occurs at intermediate ϕ values (associated with ∼60% reduced diffusivity), thus strongly supporting the earlier finding of greater energetic stabilization (Δ*T*_C_) of each polymer in the condensate at ϕ = 0.3 (Figure 2B, right panel; Figure S10). At higher ϕ, aromatic interactions limit condensate diffusivity to such an extent that the inclusion of cation−π interactions has no effect. The linkage between the effect of cation−π interactions on the energetics of the condensate and peptide diffusion within it can also be understood as an effect on the density of the condensate.

Similarly to the condensate density, the overall shape of the condensate phase may also be affected by the formation of cation−π interactions. The spherical shape of the condensate can be estimated by the shape anisotropy parameter defined as shape anistropy = . A shape anisotropy parameter value of 3 indicates that the *x*, *y*, and *z* axes of the condensate are of similar length and describes a spherical shape. The effect of cation−π interactions on the sphericity of the condensate is probed by the change in the shape anisotropy parameter upon turning on cation−π interactions.

Figure 4 suggests that a larger deviation from a spherically shaped condensate occurs at lower values for charge clustering (Figure 4A, right panel) and aromatic residue content (Figure 4B, right panel) as a consequence of the enhanced formation of cation−π interactions under these conditions. Interestingly, at a higher salt concentration, the condensate phase more closely resembles a spherical shape than it does at a low salt concentration, which can be attributed to cation−π interactions becoming more likely as electrostatic interactions weaken (Figure 3A). At a lower salt concentration, the effect of cation−π formation on condensate shape is more prominent because lowering κ significantly weakens electrostatic interactions.

To support our claim that cation−π interactions are indeed essential in stabilizing biomolecular condensates, we performed a sequence analysis of intrinsically disordered regions (IDRs) that are involved in liquid–liquid phase separation (LLPS) by examining the interplay between their charged and aromatic residues. The IDRs that are prone to phase separate via LLPS were obtained from the LLPSDB database^64^ and were compared to IDRs extracted from the human genomes. In this analysis, 12100 IDR regions from the human genome (having IUPred 2a score >0.5) and 900 IDRs from the LLPS databases have been analyzed. We have also ensured that the IDRs are sufficiently long and comprise at least 50 residues. This sequence analysis shows that enrichment of aromatic residues in the human IDRs leads toward a depletion of positive and negatively charged residues as reflected by the reduction in the mean fraction of positive or negative residues (*f*_+_ and *f*_–_, respectively) as the fraction of aromatic content, ϕ, increases (Figure 5A). This reduction of *f*_+_ and *f*_–_ as ϕ increases results in similar net charge for all IDRs which tend to be slightly negative because *f*_–_ is often greater than *f*_+_.

**Figure 5:**
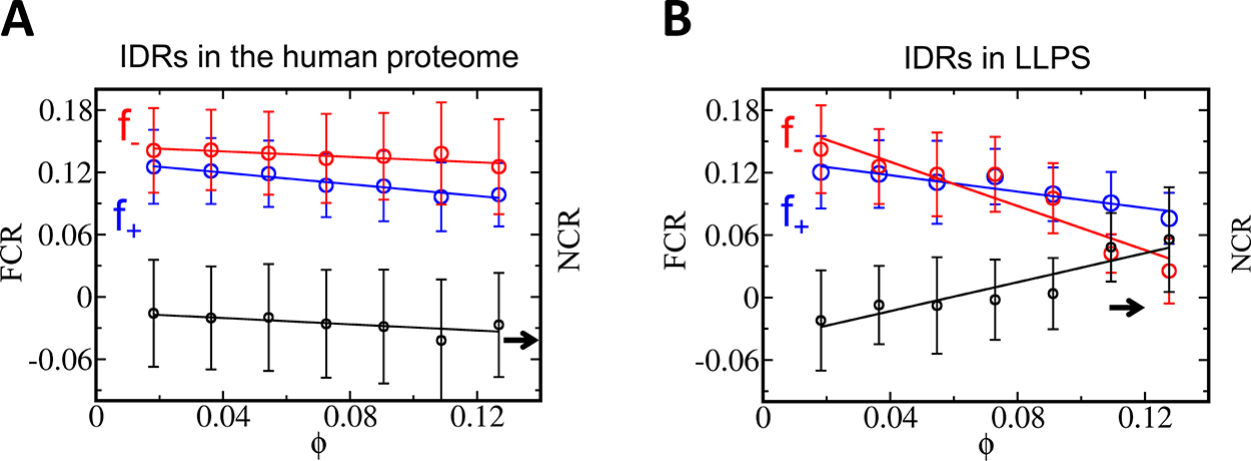
Interplay between charged and aromatic residues in IDRs. The relationship between the content of charged and aromatic residues are shown for IDRs in the human proteome (A) and for IDRs in that are prone to LLPS (obtained from the LLPSDB). (B) The fraction of positive (*f*_+_, blue circles) and negative charges (*f*_–_, red circles) are plotted against the aromatic content (ϕ) in each IDR. The IDRs are binned based on their aromatic content. For the IDRs in proteomes, the mean fraction of positive and negative charges decay linearly as the IDRs are enriched with aromatic residues. The linear fits obtained are as follows, *f*_+_ = 0.13–0.28ϕ and *f*_–_ = 0.145–0.13ϕ. The depletion of positive and negatively charged residues are nearly similar as aromatic residues enrich sequences leading to nearly polyampholyte behavior (i.e., NCR is close to zero). For IDRs that are prone to LLPS, the linear fits obtained are as follows: *f*_+_ = 0.13–0.39ϕ and *f*_–_ = 0.17–1.05ϕ. The greater depletion of the negatively charged residues than of the positively charged residues in these sequences lead toward an increase in the net positive charge of the IDR regions.

The interplay between the fraction of charged residues and the fraction of aromatic residues is different for IDRs that form LLPS. In such IDRs forming condensates, the depletion of negatively charged residues is much greater than the depletion of the positively charged residues as the IDRs become more enriched with aromatic residues (Figure 5B). This suggests a conservation of positively charged residues with aromatic residues over negatively charged residues, leading to a positive net charge of LLPS IDRs (namely, their net charge is coupled to their aromatic content). Such conserved positive charges can interact with aromatic ones under suitable conditions leading toward additional stabilization of the condensate and compensatory mechanism for the reduced stability due to electrostatics weakening.

In the current study, we built on our earlier work^44,45^ to show that the replacement of charged residues with aromatic residues in disordered peptides leads to trade-offs between short- and long-range interactions during their formation of biomolecular condensates via liquid–liquid phase separation. In particular, our simulations indicate that the formation of cation−π interactions may depend on the composition and organization of charged and aromatic residues in disordered peptide sequences and on environmental conditions (salt concentration). Bioinformatic analysis of IDR regions in LLPS prone proteins suggests a conservation of positively charged residues once aromatic residues enrich the sequences, leading toward a possibility of extensive cation−π interactions. Experimental studies of LLPS have shown that nonionic interactions are fairly important to infer stability to condensates at very high salt concentrations,^52,53^ highlighting the possibility of compensating interactions due to the loss of charge–charge interactions at high salt concentration. The strong effect salt may have on the interplay between the various molecular forces in the condensate may suggest that detailed computational studies with explicit ions and water molecules are needed to further quantify interactions between IDPs in condensates.

The formation of cation−π interactions depends on breaking charge–charge and π–π interactions. The stability of charge–charge interactions may depend on various factors, including the fraction of charged residues in the sequence (which, in the studied peptides S1–S5, was in inverse proportion to their aromatic residue content), their organizational pattern (represented by the κ value), and solvent salt concentration. We found that decreasing the fraction of charged residues in a disordered peptide, reducing its κ, or increasing the solvent salt concentration led to a loss of electrostatic interactions that reduce condensate stability. However, our simulations showed that such condensate destabilization is offset by 20–80% by the consequent increase in the formation of cation−π interactions. The gain in stability arising from increased cation−π interactions is ∼10–14 kcal/mol per peptide compared with a peptide in a condensate in which cation−π interactions do not occur. The increased stability bestowed by cation−π interactions is linked with a reduction in diffusivity of 10–60% as a function of either sequence charge clustering or aromatic content.

The existence of trade-offs^65,66^ between charge–charge, π–π, and cation−π interactions suggests that these interactions are not additive and that the loss of some can be compensated for by a gain in others. Such a mechanism suggests weak sensitivity to the loss of interactions, as they can be recovered via the formation of other interactions, as was shown here by the formation of cation−π interactions. Moreover, the trade-off mechanism may have implications for the design of synthetic condensates by leveraging the complex molecular grammar of condensate stability and dynamics to create peptides with the desired properties. Our findings suggest that the stability and dynamics of protein condensates can be tuned by manipulating the fraction of charged residues in the sequence and their clustering pattern to optimize trade-offs among charge–charge, π–π, and cation−π interactions.

## Supporting Information Available

IDR sequence information for calibration of structure-based coarse-grained model, comparison of experimentally obtained and simulated Rgs for IDR sequences (S1–S4) along variation of cation−π and aromatic strength parameters, phase diagrams for studied sequences with different amount of aromatic content along salt concentration variation, effect of sequence charge clustering on the stability of condensate phase at different salt concentrations, interchain enthalpy variation in condensates along aromatic content variation in studied sequences at different salt concentrations, interchain enthalpy variation in condensates as sequence charge clustering is perturbed in studied sequences at different salt concentrations, relative diffusivity of polymers in condensates with respect to bulk as a function of temperature for variants with differential aromatic content in sequences at different salt, relative diffusivity of polymers in condensates with respect to bulk as a function of temperature for variants with differential charge-clustering in sequences at different salt, and shape-anisotropy variation along aromatic content at different salt concentrations (PDF)
