# Supplementary material for: Cross-Talk of Cation−π Interactions with Electrostatic and Aromatic Interactions: A Salt-Dependent Trade-off in Biomolecular Condensates
Source: J Phys Chem Lett. 2023 Sep 18;14(38):8460–9. doi: 10.1021/acs.jpclett.3c01642 (PMC10544028; doi:10.1021/acs.jpclett.3c01642)
Supplement: Supplementary file 1 — jz3c01642_si_001.pdf [file jz3c01642_si_001.pdf]

Supplementary Information

Cross-Talk of Cation–π Interactions with Electrostatic and Aromatic Interactions: A Salt-Dependent Trade-off in Biomolecular Condensates

Milan Kumar Hazra and Yaakov Levy*

Department of Chemical and Structural Biology

Weizmann Institute of Science

Rehovot, 76100, Israel

*Corresponding author: Yaakov Levy, Department of Chemical and Structural Biology, Weizmann Institute of Science, Rehovot, 76100, Israel; email: Koby.Levy@weizmann.ac.il; Tel: 972-8-9344587

Proteins used to benchmark the cation-π and aromatic interaction strength (ε)

CspTm
GPGMRGKVKW FDSKKGYGFI TKDEGGDVFV HWSAIEMEGF KTLKEGQVVE FEIQEGKKGG QAAHVKV

IN
GSHCFLDGID KAQEEHEKYH SNWRAMASDF NLPPVVAKEI VASCDKCQLK GEAMHGQVDC

ProTα-C
MAHHHHHHSA ALEVLFQGPM SDAAVDTSSE ITTKDLKEKK EVVEEAENGR DAPANGNANE ENGEQEADNE VDEECEEGGE EEEEEEEGDG EEEDGDEDEE AESATGKRAA EDDEDDDVDT KKQKTDEDD

R15
KLKEANKQQN FNTGIKDFDF WLSEVEALLA SEDYGKDLAS VNNLLKKHQL LEADISAHED RLKDLNSQAD SLMTSSAFDT SQVKDKRETI NGRFQRIKSM AAARRAKLNE SHRL

R17
RLEESLEYQQ FVANVEEEEA WINEKMTLVA SEDYGDTLAA IQGLLKKHEA FETDFTVHKD RVNDVAANGE DLIKKNNHHV ENITAKMKGL KGKVSDLEKA

hCyp
SSFHRIIPGF MSQGGDFTRH NGTGGKSIYG EKFEDENFIL KHTGPGILSM ANAGPNTNGS QFFISTAKTE FLDGKHVVFG KVKEGMNIVE AMERFGSRNG KTSKKITIAD SGQLE

Protein L
MEEVTIKANL IFANGSTQTA EFKGTFEKAT SEAYAYADTL KKDNGEWTVD VADKGYTLNI KFAG

ACTR
GTQNRPLLRN SLDDLVGPPS NLEGQSDERA LLDQLHTLLS NTDATGLEEI DRALGIPELV NQGQALEPKQ D

hNHE1cdt
MVPAHKLDSP TMSRARIGSD PLAYEPKEDL PVITIDPASP QSPESVDLVN EELKGKVLGL SRDPAKVAEE DEDDDGGIMM RSKETSSPGT DDVFTPAPSD SPSSQRIQRC LSDPGPHPEP GEGEPFFPKG Q

sNase
ATSTKKLHKE PATLIKAIDG DTVKLMYKGQ PMTFRLLLVD TPETKHPKKG VEKYGPEASA FTKKMVENAK KIEVEFDKGQ RTDKYGRGLA YIYADGKMVN EALVRQGLAK VAYVYKPNNT HEQHLRKSEA QAKKEK

α-synuclein
MDVFMKGLSK AKEGVVAAAE KTKQGVAEAA GKTKEGVLYV GSKTKEGVVH GVATVAEKTK EQVTNVGGAV VTGVTAVAQK TVEGAGSIAA ATGFVKKDQL GKNEEGAPQE GILEDMPVDP DNEAYEMPSE EGYQDYEPEA

Figure S1: Comparison of radius of gyration (Rg) between simulations and experiments for different epsilon-parameter for cation-π interactions at salt concentration 20 mM while epsilon-parameter for aromatic interactions are kept fixed at 0.2 kcal/mol. The list of the proteins and their experimental Rg was obtained from literature and can be found elsewhere[1].The deviations χ^2 between simulations and experiments are shown in the top left corner of the panel. χ^2 seems to have minimum value for $\varepsilon_{Cation-\pi}$=0.1 or $\varepsilon_{Cation-\pi}$=0.2 which corresponds to highest similarity to the experimental values.

Figure S2: Comparison of radius of gyration (Rg) between simulations and experiments for different epsilon-parameter for cation-π interactions at higher salt concentration 40 mM while epsilon-parameter for aromatic interactions are kept fixed at 0.2 kcal/mol. The deviations χ^2 between simulations and experiments are shown in the top left corner of the panel. χ^2 seems to have minimum value for $\varepsilon_{Cation-\pi}$=0.2 which corresponds to highest similarity to the experimental values.

Figure S3: Comparison of radius of gyration (Rg) between simulations and experiments for sets of uniform epsilon-parameter for cation-π and aromatic interactions at salt concentration 20mM. χ^2 seems to have minimum value for ε=0.2 which corresponds to highest similarity to the experimental values.

Figure S4: Comparison of radius of gyration (Rg) between simulations and experiments for sets of same Epsilon-parameter for cation-π and aromatic interactions at salt concentration 40mM. χ^2 seems to have minimum value for ε=0.2 which corresponds to highest similarity to the experimental values.

Figure S5: Enhancement of stabilization due to cation-π crosstalk in phase behavior of condensates as a function of aromatic content and salt concentration. Phase diagrams has been plotted for sequences S1 (Panel A), S2(Panel B) and S5 (Panel C) at low salt (0.02 M, circle data points), intermediate salt (0.04 M, square data points) and high salt concentrations (0.06 M, triangle data points). While filled data points show systems with cation-π interactions void data points represent simulations without cation-π crosstalk. When the aromatic residue content in sequence is low (S1, Panel A), stability of the condensate phase comprising cation-π interactions are similar to the condensates even in the absence of cation-π interactions. Once the aromatic residue content becomes nearly equal to the fraction of positive charges in sequence (S2, Panel B), a significant enhancement is observed once the electrostatic interactions are weakened due to gradual increment of salt concentration. Once the aromatic content dominates in the sequence, additional stability obtained at different salt concentration are nearly similar.

Figure S6: Phase behavior as a function of sequence charge clustering at two different salt conditions. Panel A shows phase diagrams along variation of sequence charge clustering for

sequence set S2-S4 at lower (0.02 M) salt concentration while panel B shows the same at higher (0.06 M) salt concentration. While filled data points show systems simulated with cation-π interactions while void data points represent simulations without cation-π crosstalk. The effect of cation-π interactions on phase diagrams are manifested at low salt concentration once the tuning of electrostatics interactions are modulated by sequence charge clustering. Lower charge clustering leads toward lower electrostatic interactions and efficient cation-π stabilization of the condensate phase. At significantly higher salt concentration regime, electrostatic interactions are weakened such that effect of sequence charge clustering is equalized in sequences irrespective of charge clustering.

Figure S7: The energetic gain due to cation–π interactions for peptides S1, S2, S5, which differ with respect to aromatic content (ϕ) at low (circles, 0.02 M) and high salt concentration (triangles, 0.06 M). Maximal energetic stabilization has been obtained for ϕ=0.3 as the statistical possibility of cation-π interactions are maximum at intermediate ϕ regime.

Figure S8: Average interaction energy, a polymer faces in droplet phase due to inter-chain interactions as a function of temperatures scaled with respect to critical point of the condensates formed by sequences S1 (Panel A), S2 (Panel B), S5 (Panel C) simulated in presence of cation-π interactions (solid data points) and in absence of cation-π interactions (void data points). Circles, squares, and triangles represent data for low (0.02 M), intermediate (0.04 M), high (0.06 M) salt concentration.

Figure S9: Average interaction energy, a polymer faces in droplet phase due to inter-chain interactions, as a function of sequence charge clustering parameter (S2-S4) at low salt (panel A, Circles, 0.02 M salt concentration) and at higher salt (panel B, Triangles, 0.06 M salt concentration). Solid data points present data for systems simulated with cation-π interactions while void data points show systems in absence of cation-pi interactions. Excess stabilization of polymers in droplets are prominent when sequence charge clustering is low enough and salt concentration is also low enough. Once salt concentration is higher, all the sequences have similar stability in the condensate phase irrespective of charge clustering.

Figure S10: Reduced diffusivity in condensate phase as cation-π crosstalk increases. Translational diffusivity of polymers in the droplet phase has been compared to bulk and plotted as a function of temperatures scaled with respect to critical temperature of the condensates for S1 (Panel A), S2 (Panel B), S5 (Panel C) at low (circular data points, 0.02 M salt), intermediate (square data points, 0.04 M salt), high (triangle data points, 0.06 M salt) concentrations. When aromatic content is low in the sequence (S1), at low salt concentration, the diffusivity of the polymers in the condensate is same irrespective of cation-π crosstalk's presence or absence. But at higher salt concentration, cation-π crosstalk starts to build up and a dropdown of polymer's diffusivity is observed. When the sequence contains nearly equal number of positively charged residues and aromatic ones (Panel

B, sequence S2), the reduction of diffusivity is evident for simulations with cation-pi crosstalk with respect to the ones that are simulated in absence of cation-π interactions. At significantly higher aromatic content in the sequence (Panel C, sequence S5), aromatic π-π interactions are the dominant ones and reduction of diffusivity due to cation-π interactions are only observed at higher temperatures once significant π-π interactions are broken.

Figure S11: Sequence charge clustering affects the diffusivity of polymers in condensates by modulating inter-chain multivalent cation-π interactions. Translational diffusivity of polymers (sequence set S2-S4) in droplet phase has been compared to bulk and plotted as a function of temperature scaled to the critical temperature of the respective condensate phases for low salt concentration (Panel A, 0.02 M) and at higher salt (Panel B, 0.06 M) concentration. Reduction of diffusivity is pronounced for systems having cation-π interactions once the electrostatics is weakened through lowering the charge clustering and by enhancing the salt concentration.

Figure S12: Droplets turn spherical once cation-π cross talks start to build up. Shape anisotropy of droplets has been shown along temperature scaled with respect to critical point of the respective condensate phase for sequences having lower (Panel A, ϕ=0.1), intermediate (Panel B, ϕ=0.3), higher salt concentration (Panel C, ϕ=0.6). Each panel consists of data for lower (circle data points, 0.02 M salt), intermediate (square data points, 0.04 M salt) and high salt concentration (triangle data points, 0.06 M salt). While filled data points show systems with cation-π interactions, void data points represent simulations without cation-π crosstalk. When aromatic content in sequence is low (Panel A, S1, ϕ=0.1), intermediate and higher salt concentrations help to weaken the electrostatic interactions and facilitate cation- π resulting in a dense more spherical condensate with respect to the condensate formed in absence of cation- π crosstalk. While intermediate aromatic content in sequence (Panel B, S2, ϕ=0.3), maximizes the cation-π possibility at higher salt resulting in a prominent decrease in condensate asphericity. Once the aromatic content in sequence reaches a threshold π-π interactions dominate and change in droplet shape at different salt concentrations are negligible with respect to systems simulated in absence of cation-π interactions.

(1) Dignon, G. L.; Zheng, W.; Kim, Y. C.; Best, R. B.; Mittal, J. Sequence Determinants of Protein Phase Behavior from a Coarse-Grained Model. *PLoS Comput Biol* **2018**, *14* (1). https://doi.org/10.1371/journal.pcbi.1005941.

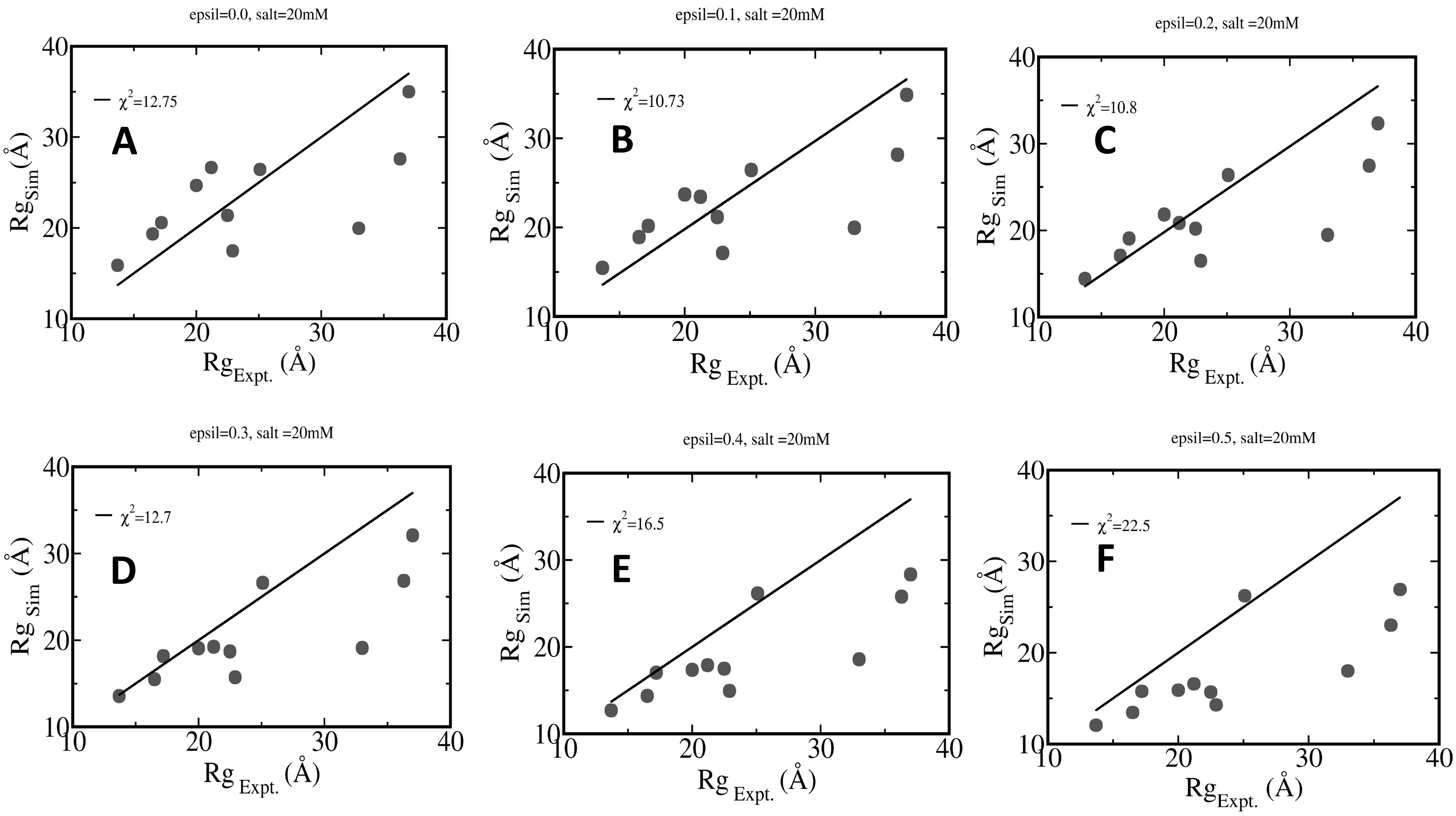

epsil=0.0, salt=20mM
χ2=12.75
A
RgSim (Å)
RgExpt. (Å)
epsil=0.1, salt =20mM
χ2=10.73
B
Rg Sim (Å)
Rg Expt. (Å)
epsil=0.2, salt =20mM
χ2=10.8
C
Rg Sim (Å)
Rg Expt. (Å)
epsil=0.3, salt =20mM
χ2=12.7
D
Rg Sim (Å)
Rg Expt. (Å)
epsil=0.4, salt =20mM
χ2=16.5
E
Rg Sim (Å)
Rg Expt. (Å)
epsil=0.5, salt=20mM
χ2=22.5
F
RgSim (Å)
RgExpt. (Å)

Fig. 1

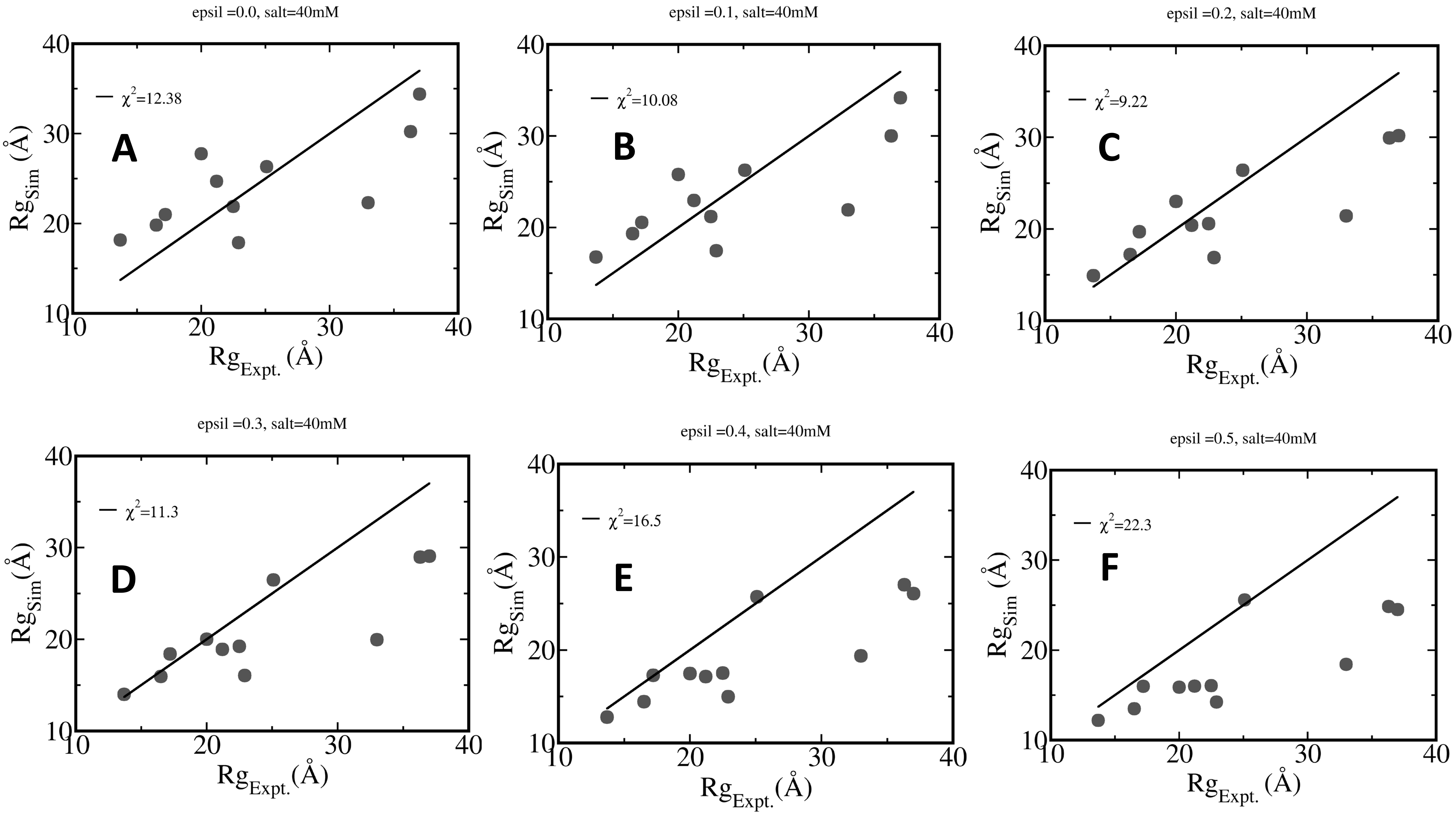
epsil =0.0, salt=40mM
χ²=12.38
A
epsil =0.1, salt=40mM
χ²=10.08
B
epsil =0.2, salt=40mM
χ²=9.22
C
epsil =0.3, salt=40mM
χ²=11.3
D
epsil =0.4, salt=40mM
χ²=16.5
E
epsil =0.5, salt=40mM
χ²=22.3
F
Rg_Sim (Å)
Rg_Expt. (Å)
10
20
30
40

Fig. 2

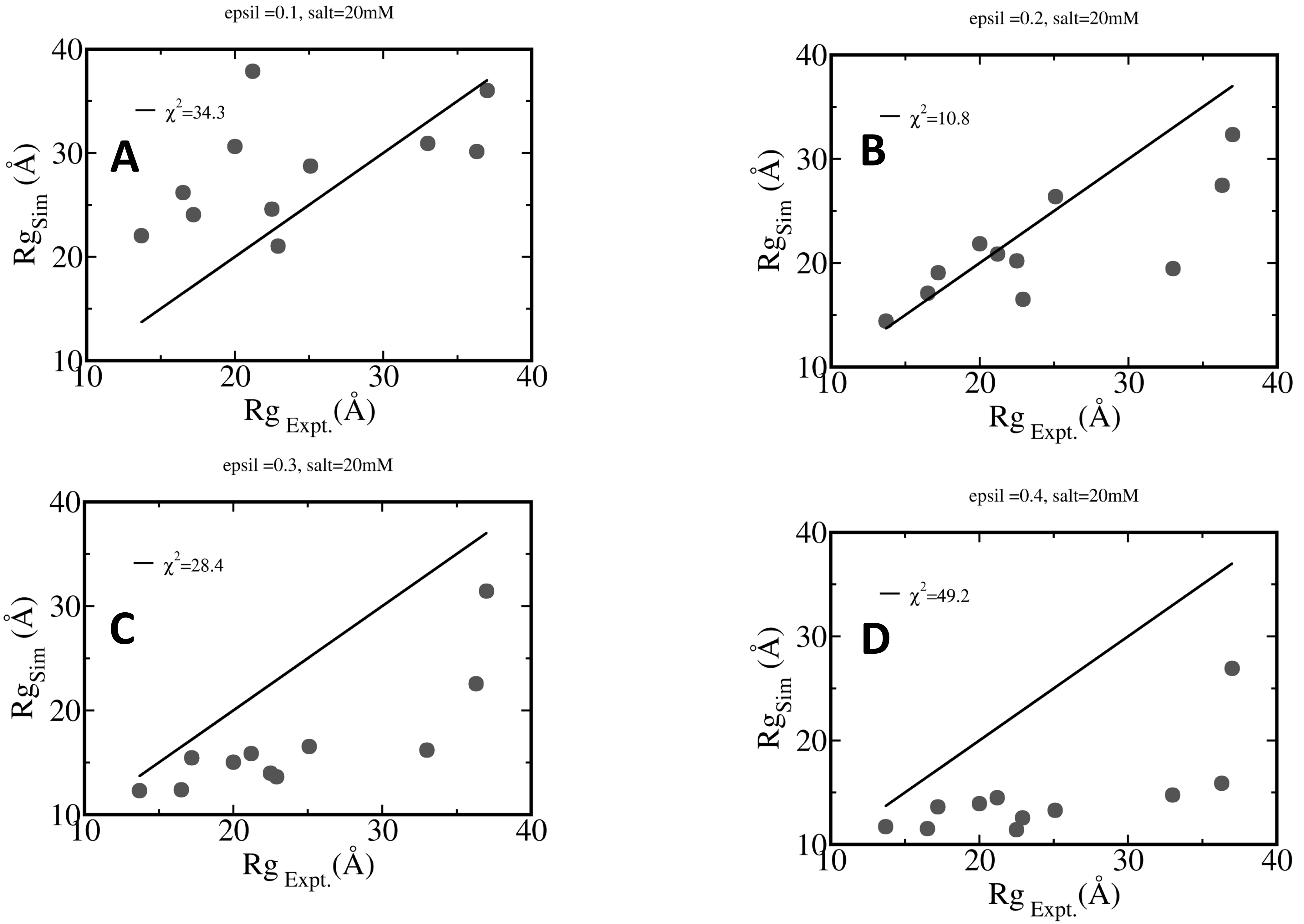
epsil =0.1, salt=20mM
χ^2=34.3
A
epsil =0.2, salt=20mM
χ^2=10.8
B
epsil =0.3, salt=20mM
χ^2=28.4
C
epsil =0.4, salt=20mM
χ^2=49.2
D
Rg_{Sim} (Å)
$Rg_{Expt.}$(Å)
10
20
30
40

Fig. 3

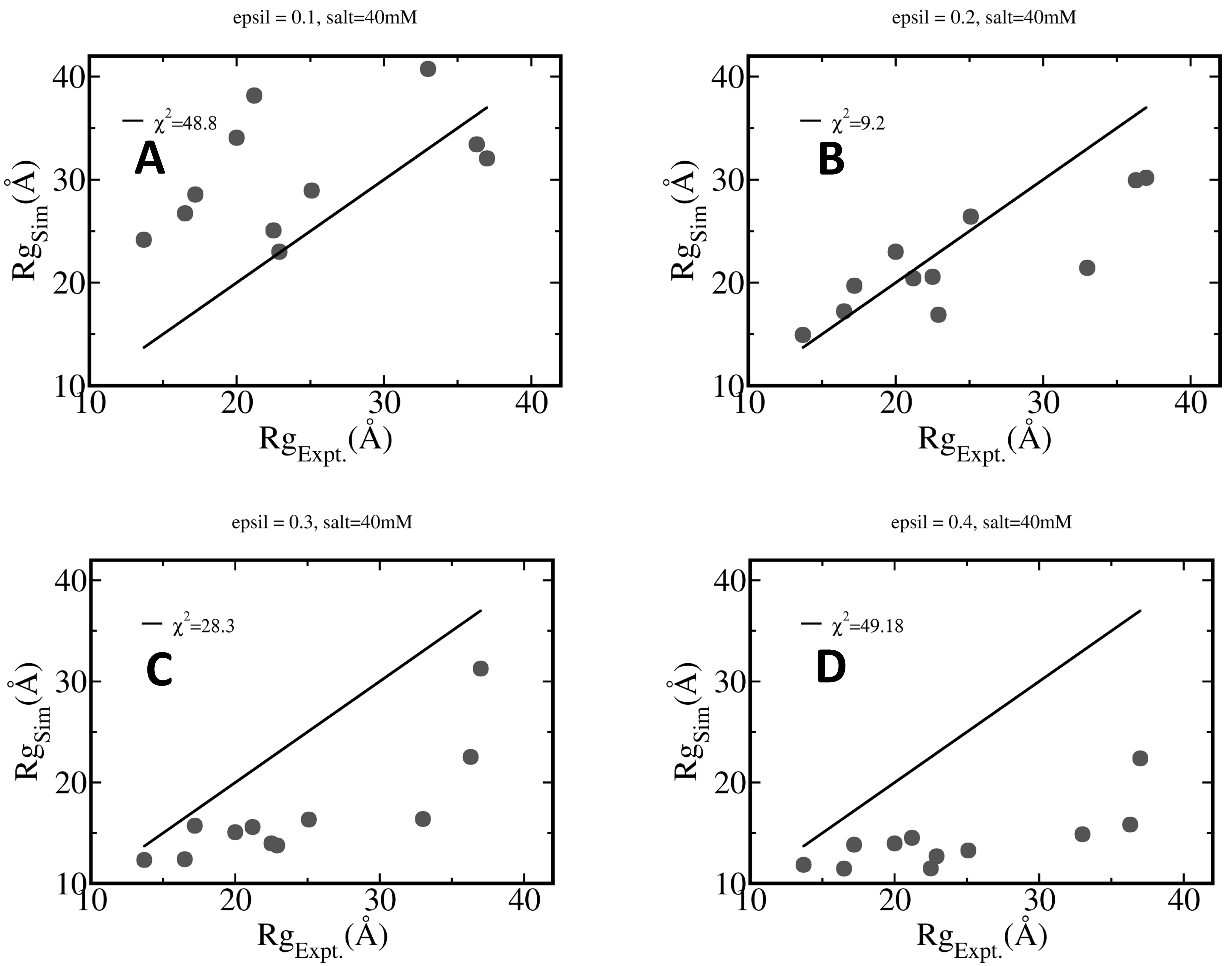

epsil = 0.1, salt=40mM
χ^2=48.8
A
epsil = 0.2, salt=40mM
χ^2=9.2
B
epsil = 0.3, salt=40mM
χ^2=28.3
C
epsil = 0.4, salt=40mM
χ^2=49.18
D
Rg_{Sim}(Å)
$Rg_{Expt.}$(Å)
10
20
30
40

Fig. 4

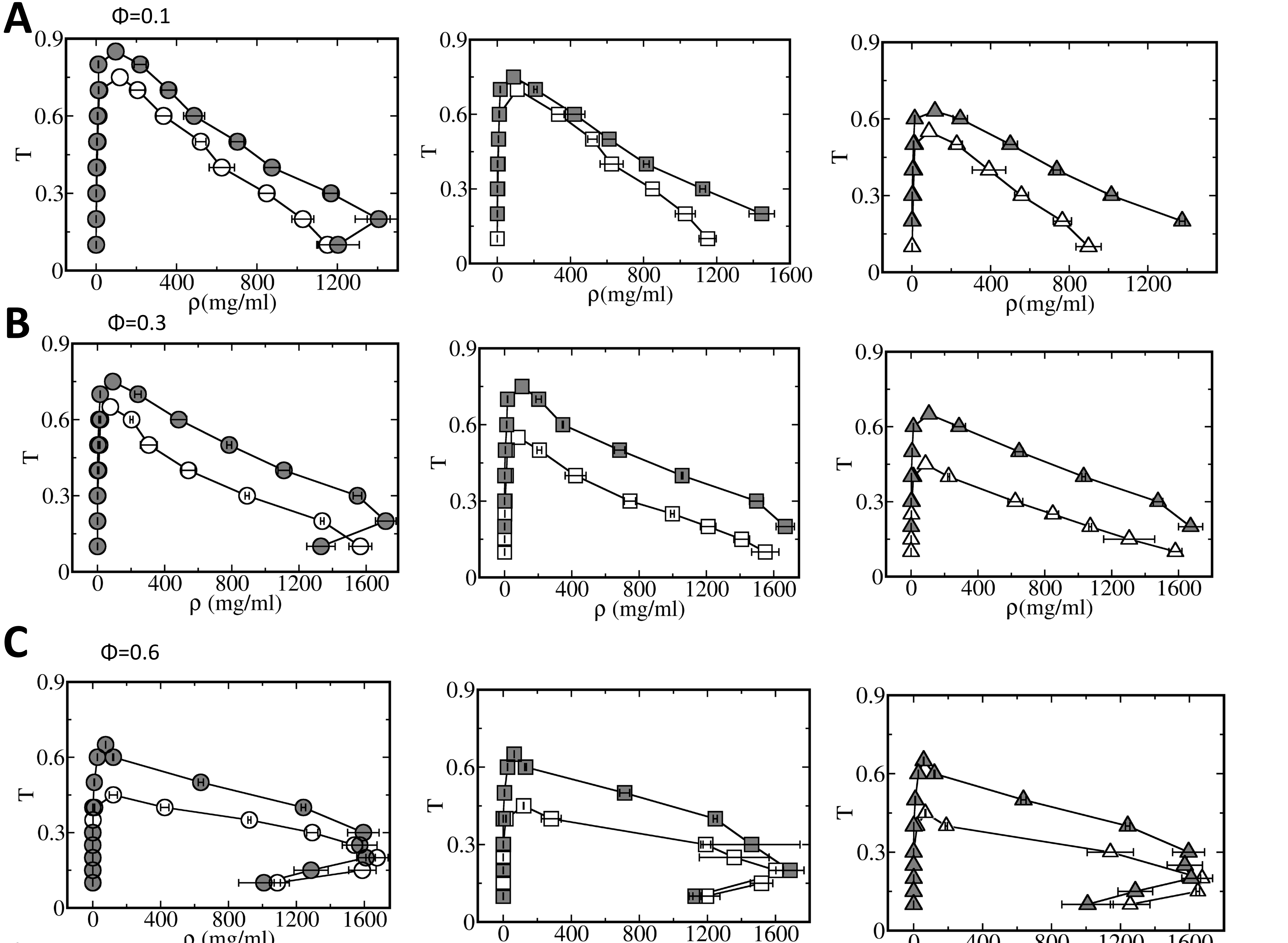

Fig. 5

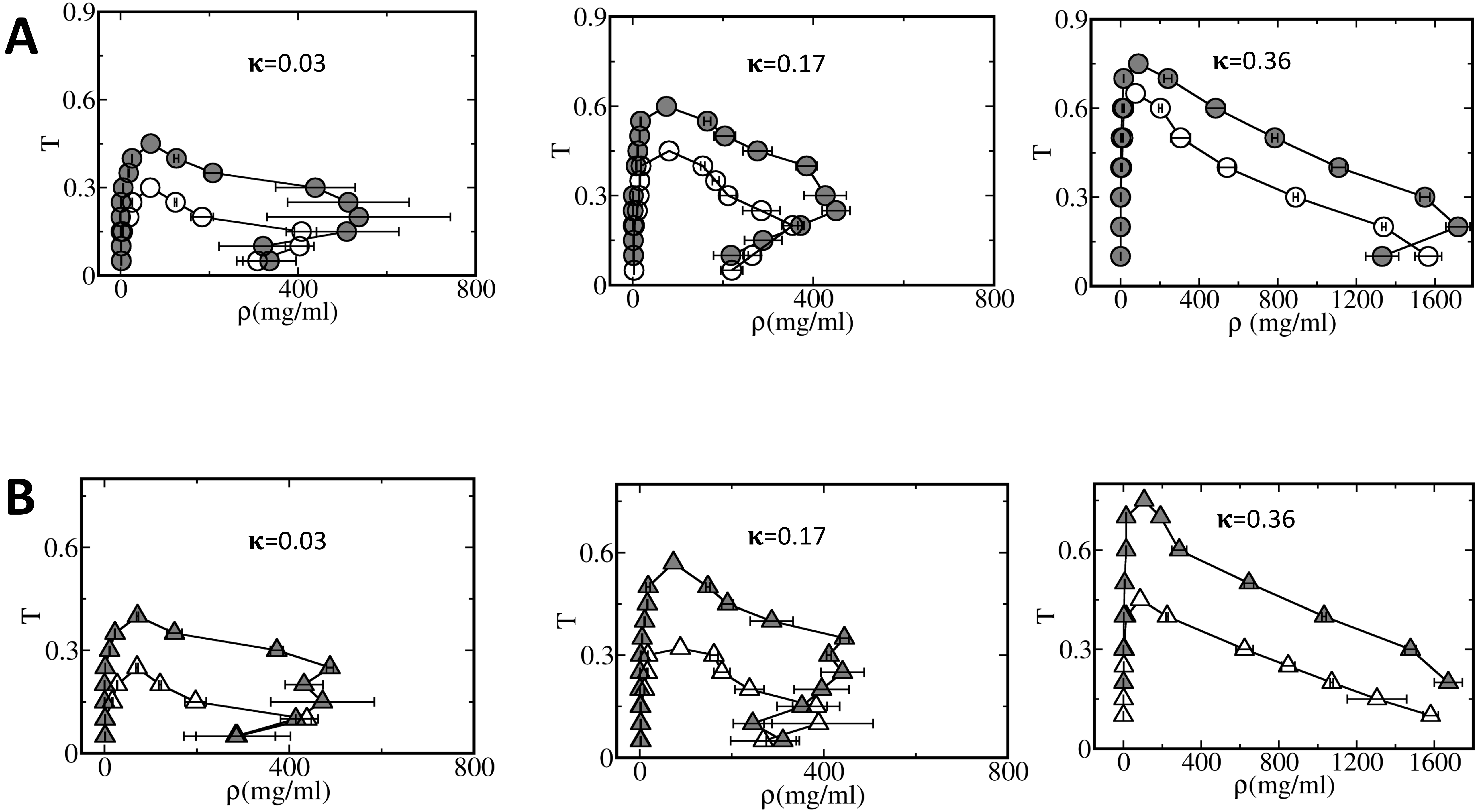

Fig. 6

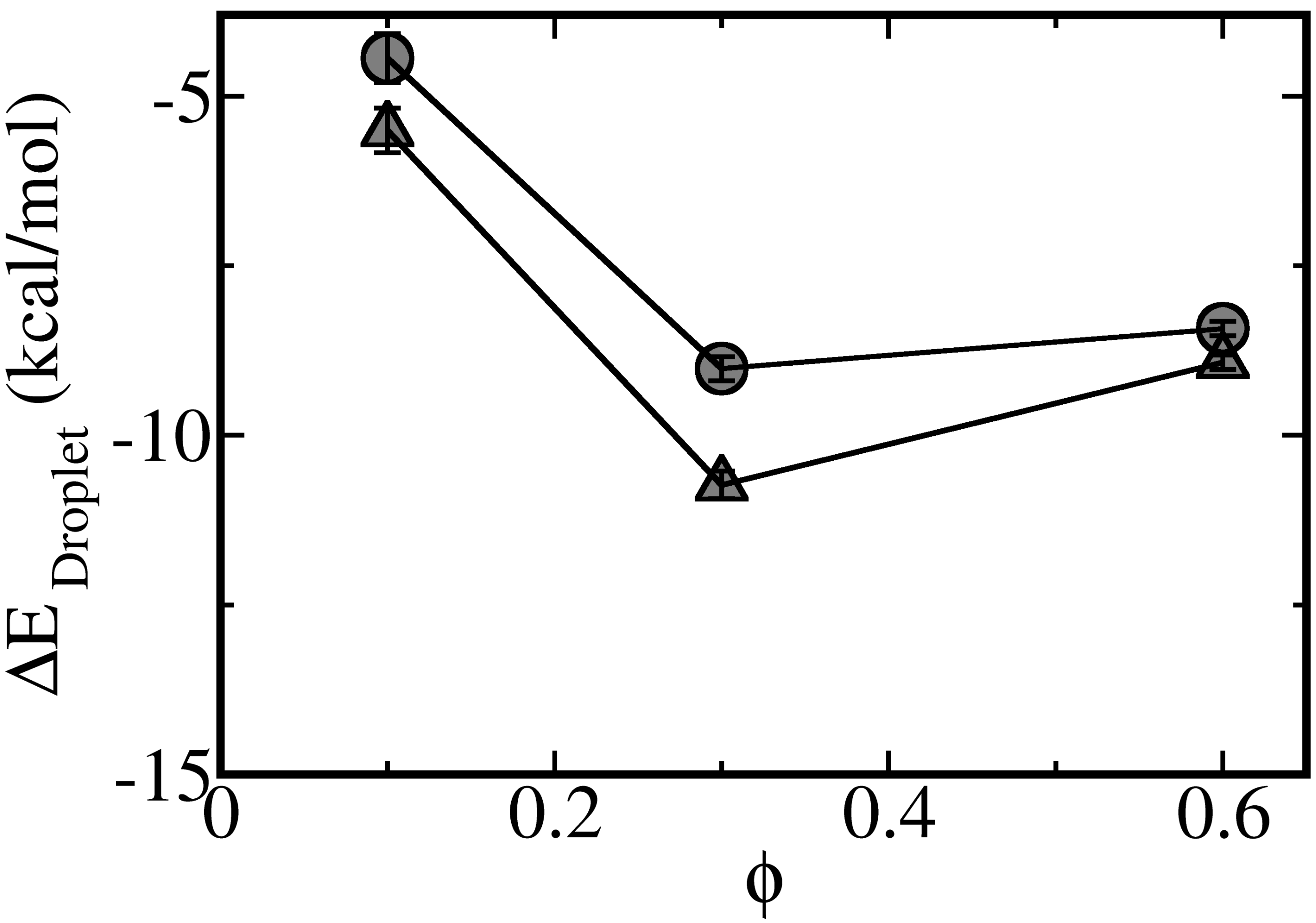

Fig. 7

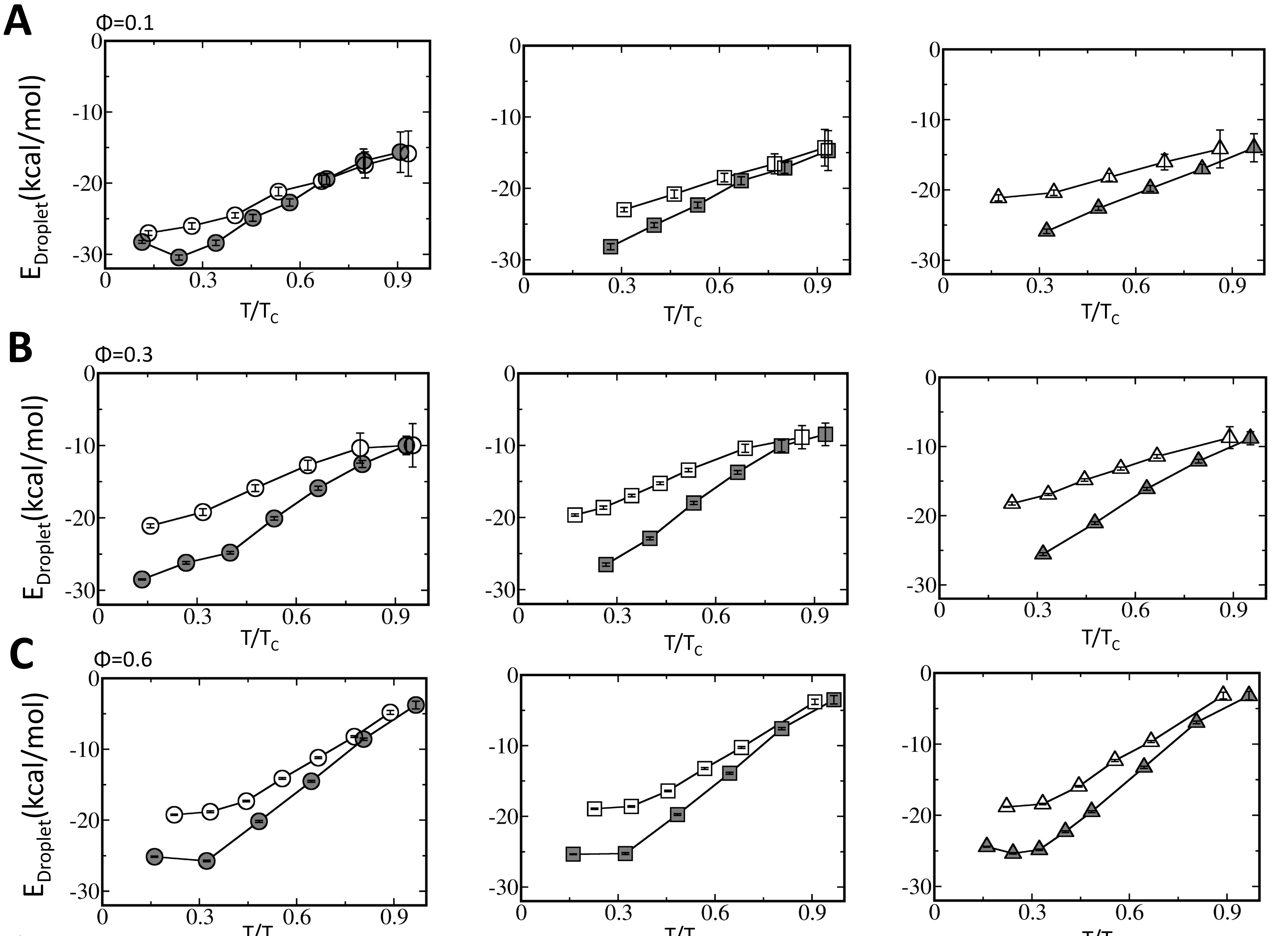

Fig. 8

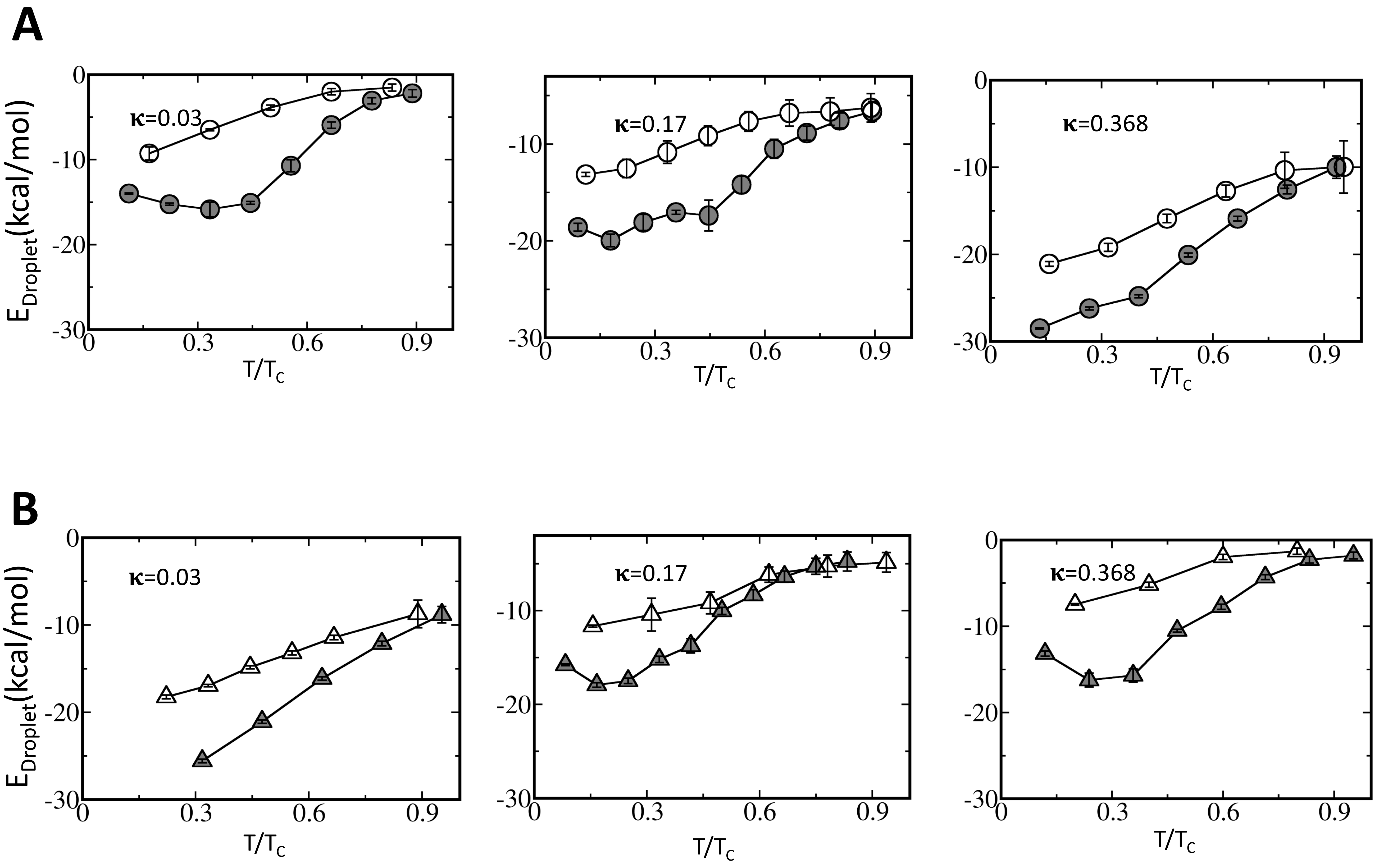

Fig. 9

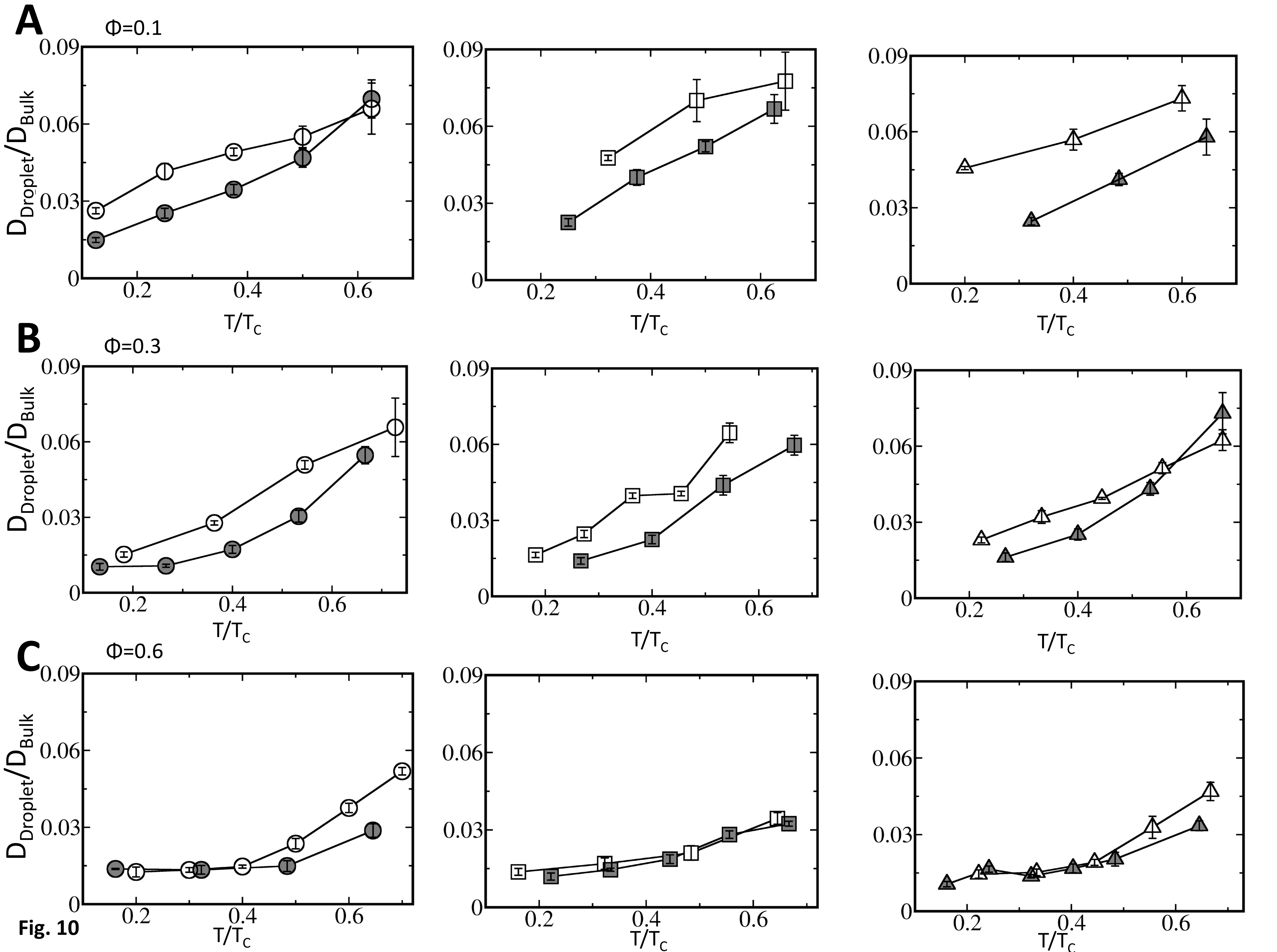

Fig. 10

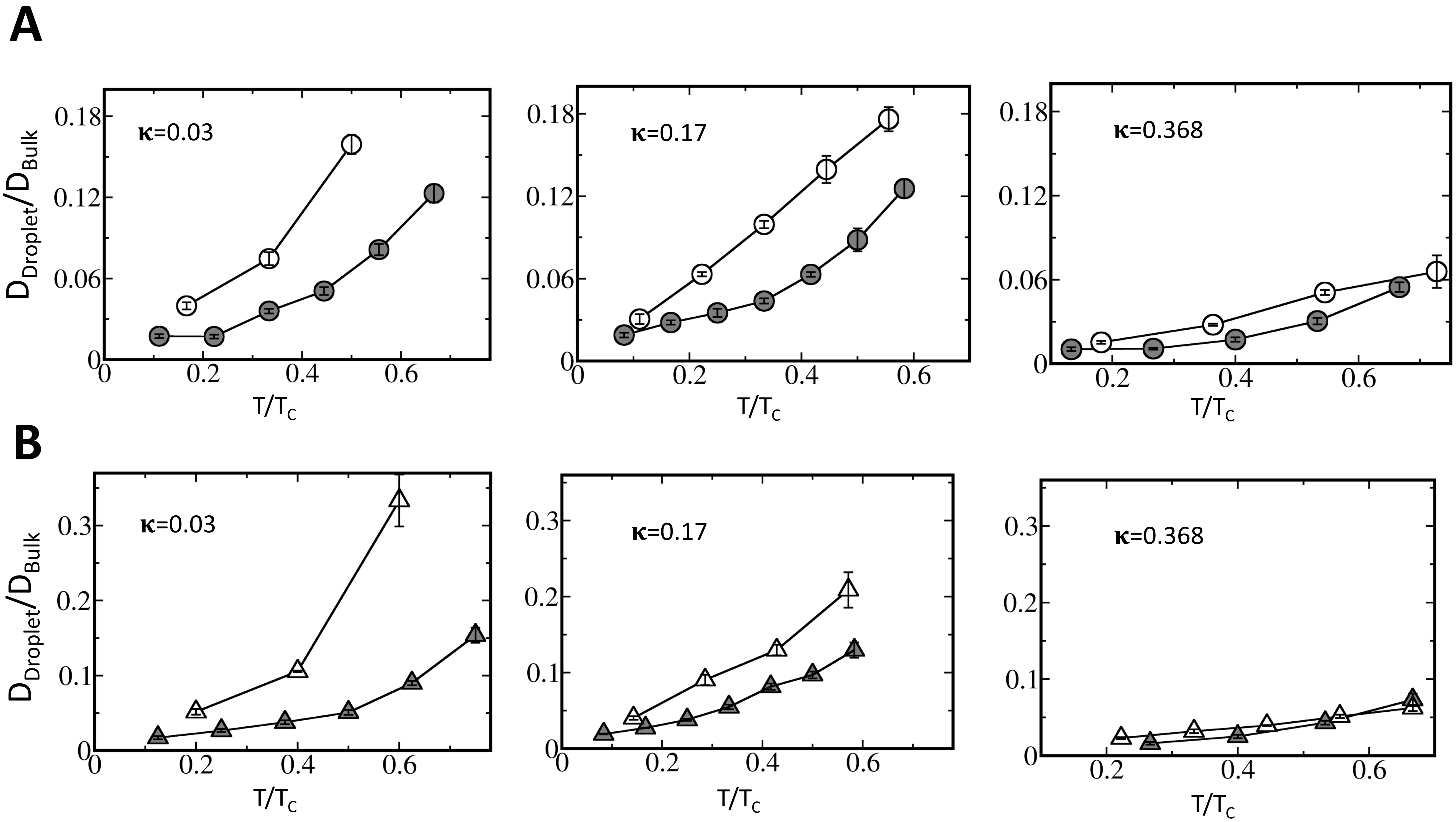
A
B
κ=0.03
κ=0.17
κ=0.368
κ=0.03
κ=0.17
κ=0.368
$D_{Droplet}/D_{Bulk}$
$D_{Droplet}/D_{Bulk}$
T/T_C
0
0.06
0.12
0.18
0.1
0.2
0.3
0.2
0.4
0.6

Fig. 11

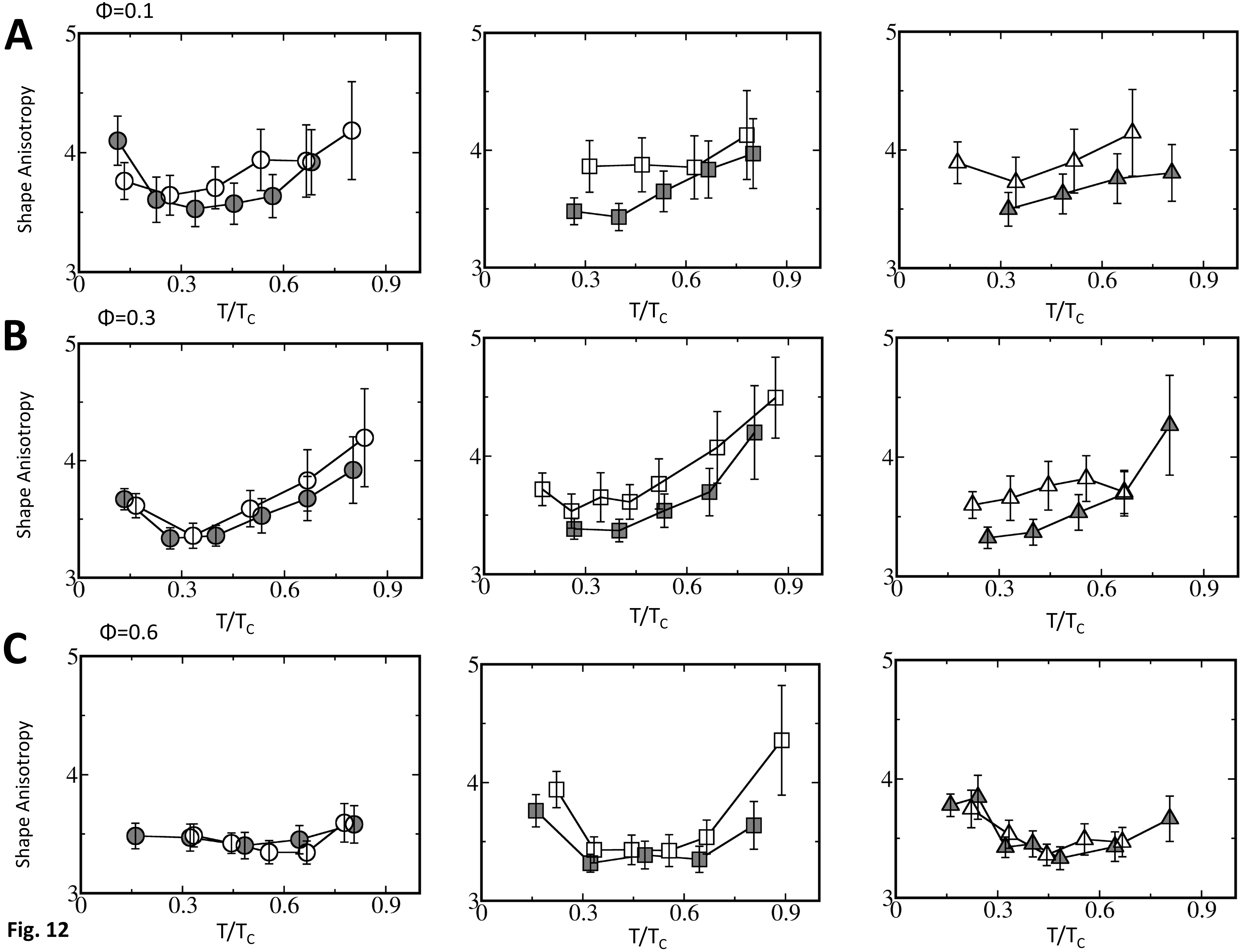

Fig. 12
